# The implications of uncertainties on global lithium resources availability estimations

**DOI:** 10.1016/j.isci.2026.114629

**Published:** 2026-01-06

**Authors:** Dongping Zhou, Steve Pye, Brunilde Verrier, Paul Dodds

**Affiliations:** 1 UCL Energy Institute, University College London, London WC1H 0NN, UK; 2 UCL Institute for Sustainable Resources, University College London, London WC1H 0NN, UK

**Keywords:** energy resources, applied sciences, economics

## Abstract

The accelerating energy transition, driven by decarbonization goals and energy security concerns, is intensifying global lithium demand. However, few studies assess whether supply can meet this demand under uncertainty. This study examines long-term lithium availability by constructing a global bottom-up deposit database and generating a cumulative availability curve (CAC). A six-step methodology combining Monte Carlo and depletion curve analyses estimates 35.5 Mt of lithium, with costs from 1,194 to 31,590 USD/t LCE (2023). Current supply is dominated by low-cost Australian ore, while future production may depend more on brine. Comparison with IEA demand scenarios indicates that conservative assumptions could lead to shortfalls, whereas meeting ambitious climate goals may entail higher costs. Sensitivity analysis shows that physical and technical uncertainties exert the greatest influence. These findings underscore the need for timely investment in deposits and recycling infrastructure.

## Highlights

• A global lithium database was constructed covering 188 deposits
• Key uncertainties in lithium cumulative availability curves are classified
• Recoverable lithium resources are estimated at 35.5 million tonnes
• Lithium all-in costs range from 1,194 to 31,590 USD/t LCE

## Graphical abstract

**Figure:**
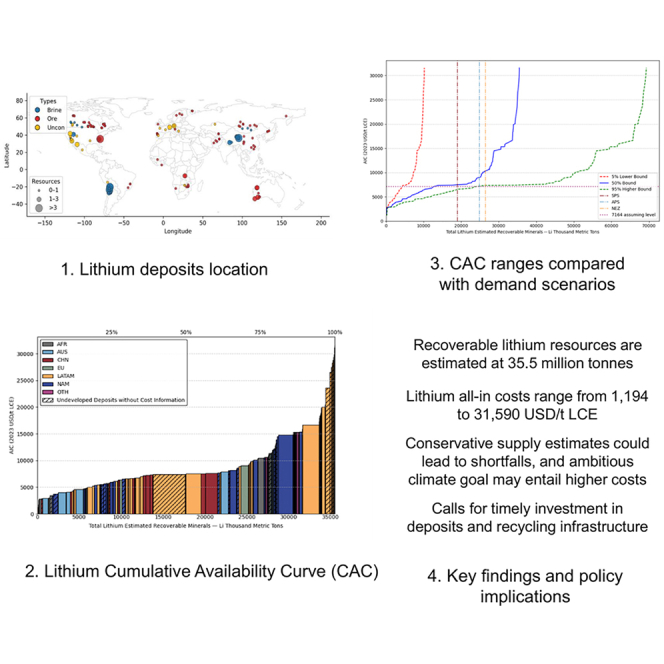

## Introduction

The consumption of fossil fuels has been pivotal in driving global industrialization and economic growth.^1^^,^^2^ However, this has also led to a sharp increase in CO_2_ emissions, which threatens the Earth’s environmental system.^3^^,^^4^ A global shift to renewable energy is essential to decouple economic growth from carbon emissions, but it also drives a substantial rise in demand for critical minerals.^5^^,^^6^^,^^7^ Among energy transition minerals, lithium holds a vital position owing to its unparalleled properties for electrochemical energy storage.^8^ Its demand is primarily driven by two requirements: the need to buffer the intermittency of renewable energies,^9^^,^^10^ and the widespread adoption of electric vehicles equipped with power batteries.^11^ Various energy scenarios project that by 2050 the global lithium demand could increase by around 20 to 35 times compared to 2020 levels,^7^^,^^12^^,^^13^^,^^14^ with more ambitious decarbonization pathways driving demand toward the upper bound. Ensuring a stable lithium supply in the coming decades is therefore crucial to meeting global climate goals.

Most lithium resource studies estimate that the available lithium vastly exceeds even the highest demand forecast.^15^^,^^16^^,^^17^ However, global supply chains remain fragile and many countries list lithium as a critical mineral.^18^^,^^19^^,^^20^ First, regarding concentration risk, lithium extraction and refining are dominated by a small number of producers, with the top three producing countries accounting for roughly 85% of mine output and 96% of refining capacity.^21^ Second, recycling constraints mean that lithium’s end-of-life recovery rate is currently only about 3%, leaving the system ill-prepared for any major supply shock.^7^ Third, price volatility over the past decade have seen lithium price swings through a cycle by nearly a factor of ten, resulting in severe disruptions to the supply system.^22^ These factors highlight the need to examine lithium availability more closely, not just its resource potential, but also its geographic distribution and relevant supply costs.

It is observable that research in lithium resources has been intensifying. However, most existing studies are primarily demand-driven, commonly using material flow analysis (MFA), with transparent mass-balance accounting. Among these, some studies adopt a national perspective, for example focus on China^23^^,^^24^^,^^25^ and on the United Kingdom,^26^ while others examine the issue from a global standpoint.^27^^,^^28^ In addition, system dynamics (SDs) represents another important methodological approach, enabling the exploration of complex feedback loops and temporal interdependencies within the lithium supply chain.^29^^,^^30^ However, it often abstracts away cross-sectoral drivers, such as policy regimes, emissions constraints, trade and (Environmental, Social, and Governance) ESG frictions, and is not guided by a cost-optimal consideration. Besides, hybrid models that combine MFA and SD have been developed, aiming to leverage the strength of both approaches.^31^^,^^32^ Yet the supply-side representation in these studies are often overly simplified. For instance, Lähdesmäki et al. (2003)^33^ relies on US Geological Survey (USGS) reserves and production statistics, Pratap et al. (2024)^30^ draws on projections from International Energy Agency (IEA) reports, and Sverdrup (2016)^34^ synthesizes data from a serval of literature sources. The coarse granularity of these input data, results in estimates that are prone to being less reliable and are inadequate as inputs to more comprehensive systems research, such as with Integrated Assessment Models.^35^

In contrast to the flourishing body of research projecting lithium demand, exploration on the supply side remains limited. Much of the existing work adopts a traditional mineralogical focus, examining source classifications,^36^^,^^37^ extraction methods,^38^ and resource estimates.^16^^,^^39^^,^^40^ However, research explicitly linking these aspects with production costs remains scarce. Cumulative availability curve (CAC) analysis is a widely used tool that combines data on the physical abundance of resources with economic factors to assess mineral availability. While the CAC framework has been used to long-term availability and depletion for lithium,^41^^,^^42^^,^^43^ in contrast to the more mature oil and natural gas sectors,^44^^,^^45^ these cost curve studies have yet to rigorously evaluate the effects of underlying uncertainties on their estimates.

Uncertainty is inherent in estimates of resource availability, making the design of effective policy around resource management particularly challenging. On such uncertainties a substantial literature has emerged.^46^^,^^47^^,^^48^ One strand attributes uncertainty to inadequate information (epistemic uncertainty): Stirling (2010)^49^ conceptualizes “alternative states of incomplete knowledge” along two dimensions: incomplete knowledge of outcomes and incomplete knowledge of probabilities. In contrast, Walker et al. (2003)^50^ emphasize that gaining knowledge may sometimes widen the bounds of uncertainty, either by revealing limitations in understanding or by exposing greater system complexity. Building on a typological approach, Walker et al. (2003)^50^ classify uncertainty along three orthogonal dimensions: location, level, and nature. The category of nature encompasses epistemic and variability (aleatory) uncertainty. Building on such schemas, Speirs et al., (2015)^17^ expand the lens to five domains relevant to resource availability: physical, technical, economic, socio-political, and sustainability. For resource availability estimate application, McGlade (2014)^44^ provides a systematic treatment of supply-side data for oil and natural gas, grouping into repeatable epistemic, non-repeatable epistemic, terminology uncertainty, simplifying assumptions, and random macroscopic uncertainties, with particular attention to terminological inconsistencies. At the deposit scale, Lindi et al. (2024)^51^ highlight how uncertainties in mineral resources estimation originate at early exploration and then propagate and accumulate through project stages.

These contributions collectively show both the pervasiveness of uncertainty and the overlaps between categories. In this article, the focus is on lithium and the construction of CAC. For analytical clarity, uncertainties are organized into three classes: (1) terminology uncertainty, concerning definitions and the mapping between lithium resource categories and production cost; (2) uncertainty related to CAC construction, encompassing parameter choices and modeling assumptions; and (3) uncertainty related to CAC application originate from external factors influencing resource demand and extraction. The relevant information has been summarized in Table 1. Understanding these three types of uncertainty, and integrating the first two into the CAC construction framework while considering the third in subsequent applications, enables the framework to support robust analysis and inform policy design that aligns low-carbon objectives with economic and energy-security considerations.

**Table 1:** Uncertainty classification related to CAC

Uncertainty classification	Explanation	Sub-class	Example	Assess methods (Details in Method details)
Terminology uncertainty	Inconsistent definitions or comparison of disparate terms	–	Resource and reserves, production cost	Terminology clarification and ranking, Monte Carlo analysis, sensitivity analysis
Uncertainty related to CAC construction	Arises from translating geological, engineering and economic assumptions into CAC.	Physical	Volumetric, grade, exploration status	Triangular distribution, factor α with normal distribution, Monte Carlo analysis, sensitivity analysis
–	–	Technical	Recovery rate, production status	Factor β with normal distribution, Monte Carlo analysis, sensitivity analysis
–	–	Economic	Production cost	Depletion curve
Uncertainty related to CAC application	Emerges when a CAC is applied, driven by external factors that affect lithium value chain	ESG	Social license, pollution fee, protest	Scenarios analysis, integrated assessment model
–	–	Recycling	Recycling cost, recycling capacity	Scenarios analysis, integrated assessment model
–	–	Geopolitical	Export control, tariffs	Scenarios analysis, integrated assessment model
–	–	Future random	Technological breakthrough, substitute material	Scenarios analysis, integrated assessment model

Explanation, sub-class, example, and assess methods.

Hence, this study presents the development of a conceptually consistent lithium deposit database, using it to construct a lithium CAC, and to assess the implications of uncertainties on future supply and costs. For deposits lacking production cost data, an estimate method is applied based on regional depletion curve similarity to infer their cost profiles. The resulting insights provide a more robust approach to lithium resource estimation and costs analysis. Importantly, they also serve as a critical first step toward integrating lithium supply chain into energy system models, similar to approaches long established for base minerals,^52^^,^^53^ thereby enabling a deeper exploration of future supply constraints and costs for the global clean energy transition.

## Results and discussion

### Resources database

This study compiles data on 188 lithium deposits, consisting of 58 brine, 106 ore, and 24 unconventional, through three data sources: global and national datasets from official organizations, academic literature, and company reports. Among them, 121 include cost-related information, while 66 do not. S&P^54^ indicates that 35 deposits were operational in 2023. The detailed information provided in Tables S3–S9.

**Figure 1:**
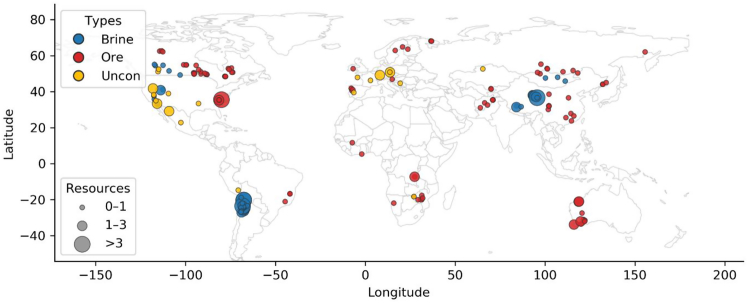
Lithium deposits location and resource amount information Color represents deposit types, Circle size represents deposit average resources amount (million metric ton lithium content); the biggest circle represents resources mean over 3 Mt Li, the mid-size represents resources mean among 1–3 Mt Li, and the smallest represents resource mean lower 1 Mt Li.

These data are held on a deposit and country basis (Figure 1). To better assess further analysis, the dataset is then further aggregated by region, reflecting global differences in economic development and lithium extraction condition. The world is divided into seven major regions: Africa (AFR), Australia (AUS), China (CHN), Europe (EU), Latin America (LATAM), North America (NAM), and other (OTH), which focuses on the Russia and central Asian countries. Ore deposits occur across all seven regions, whereas brine deposits are confined to CHN, EU, LATAM, and NAM. Unconventional sources are not regionally disaggregated due to limited data and their early-stage, exploratory status. This classification produces 12 distinct analytical groups in Table S10.

**Figure 2:**
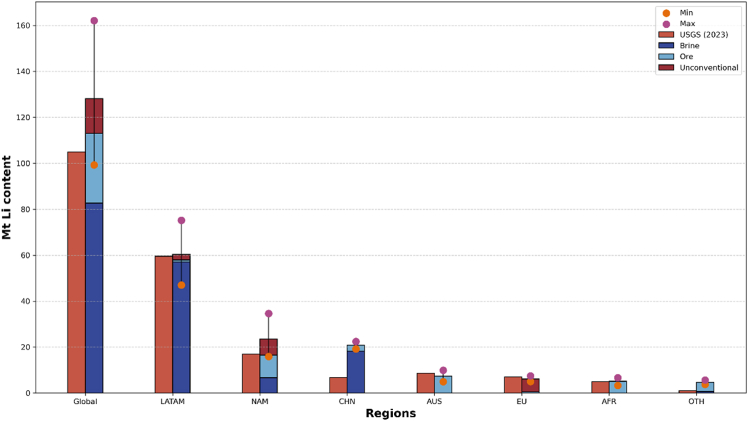
Global and regional lithium resource estimates compared to USGS The first bar represents lithium resource estimates reported by the USGS (2023), while the second bar represents the average (mean) lithium resource estimates derived in this study for each region. The lower and upper points indicate the aggregated minimum and maximum estimates, respectively, compiled across all resource types and data sources considered. Estimates are expressed in Mt of lithium content.

The database resources information are summarized and visualized in Figure 2 to offer an overview of the raw estimated resources data. At the global level, estimated lithium resources in the database show substantial variability, ranging from 99.28 to 162.16 million metric tons lithium content (Mt Li), with a mean value of 128.25 Mt Li, slightly exceeding the USGS estimate for 2023 (105 Mt Li).^55^ This spread is primarily driven by uncertainties in LATAM and NAM. In LATAM, most variation arises from estimates of Boliva’s Salar de Uyuni, one of the largest salt flats. Despite its vast theoretical capacity, the commercial development of Uyuni has progressed slowly, due to low lithium grade, a high Mg/Li ratio complicating extraction, and Bolivia’s state-led development approach, which has created persistent negotiation challenges with foreign investors.^56^^,^^57^ As for NAM, discrepancies with USGS figure mainly involve North Carolina’s Kings Mountain Belt, where differing naming conventions may have led to double counting, and Nevada’s Kings Valley projects, an unconventional source still in early exploration. As such, resource estimates for Kings Valley have fluctuated considerably over time, evolving geological and technical assessments.^36^^,^^41^^,^^58^^,^^59^

Notable differences from USGS data also emerge in brine estimates for CHN. For CHN, the main divergence involves Qarhan Salt Lake, a supergiant brine deposit comparable to Bolivia’s Uyuni. Its vast extent results in considerable variation in lithium concentration by sampling location,^60^^,^^61^ and amplify uncertainty when extrapolating total resources.^51^ Moreover, Qarhan’s location in an environmentally sensitive area means current or potential regulations add further uncertainty.^62^^,^^63^

### Global lithium cumulative availability curve

#### Global CAC for lithium from brine

**Figure 3:**
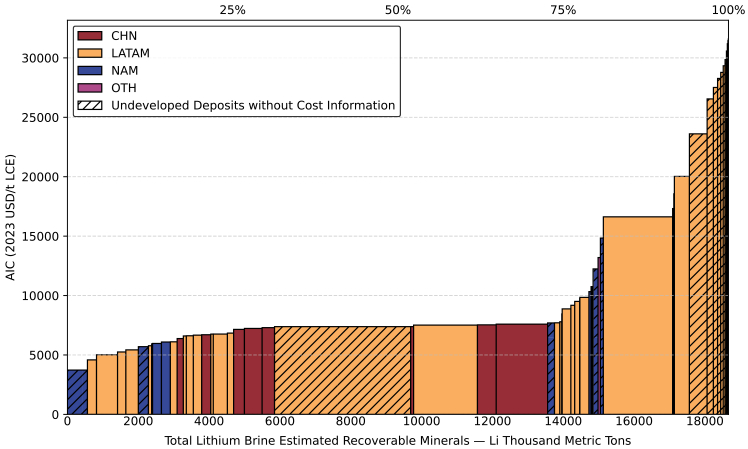
Global lithium CAC from brine separated by region See also Figures S2A, S3, and S6.

According to Figure 3, the globally lithium estimated recoverable minerals (ERMs) from brine sources amounts to 18,666 thousand metric tons of lithium content (kt Li), with most of these resources concentrated in the LATAM region. The associated all-in cost (AIC) spans a wide range, from 3,728 to 31,589 USD per metric ton of lithium carbonate equivalent, expressed in 2023 prices (USD/t LCE [2023]), reflecting substantial cost variability across deposits. Globally, approximately 10% of brine-based resources (around 1,800 kt Li) have an AIC below 5,300 USD/t LCE (2023). Up to 75% of the ERM (14,000 kt Li) falls below 8,500 USD/t LCE (2023). Across all regions, operating brine projects generally exhibit lower AICs than this range, apart from two projects in Latin America whose AICs exceed 16,500 and 20,000 USD/t LCE (2023), respectively (Figure S2).

Further analysis reveals that these two deposits are in Chile’s Atacama region. The unusually excessive costs are attributable to exceptionally high royalty payments. Unlike other lithium producing regions where royalty rates as share of revenues are relatively low, such as Argentina (3%) and AUS (5%), Chile imposes variable royalties UP to 40%, depending on market price.^64^ In 2023, this resulted in royalty payments of approximately 10,000 USD/t LCE (2023) for Atacama’s projects.^54^ At the historical price peak of lithium carbonate in 2023, supply demand imbalances allowed even high-cost projects, such as those burdened by Chile’s elevated royalty rates, to remain profitable. However, as global production capacity increases and market competition intensifies, Chile’s comparatively high royalty burden is expected to constrain the international competitiveness of its lithium products. This high-cost tail also shapes the regional curve, making the tail-end of LATAM’s CAC extremely expensive and signaling potential exposure to elevated royalty-cost risk.

#### Global CAC for lithium from ore

**Figure 4:**
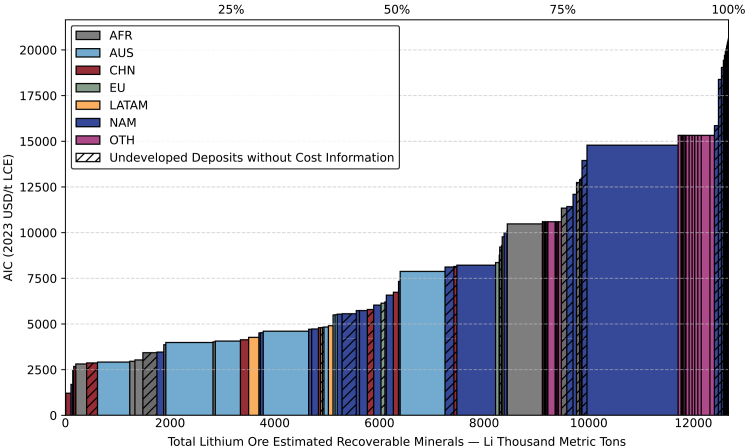
Global lithium CAC from ore separated by region See also Figures S2B, S4, and S7.

According to Figure 4, the globally lithium ERM from ore sources is 12,670 kt Li, roughly two-thirds of that from brine sources. Although ore accounts for only 37% of total resource volumes, its recoverable proportion is substantially higher. Thus, while brine dominates in terms of overall resource abundance, ore deposits are crucial for actual supply due to their superior extraction efficiency.

Ore-based lithium projects exhibit a cost range from 1,194 to 20,618 USD/t LCE (2023). Unlike brine sources, which follow a steeper cost increase in the upper quartile, the ore-based cumulative cost distribution demonstrates a more gradual and consistent upward trend. AUS holds a dominant position in ore lithium supply, accounting for most operating mines. These projects benefit from relatively low costs, with most falling within the 3,000–4,500 USD/t LCE (2023) range. AUS’s advantage stems from its well-established mining infrastructure, political stability, and consistent regulatory environment, all of which support large-scale, capital-efficient operations and sustained global exports.^65^ This stable and sustained development strategy offers a valuable mode for other resource-rich countries, helping them avoid the pitfalls of the so-called “resource curse.”

AFR, while not yet an influential player, hosts several low-cost, underdeveloped ore resources that could play a growing role in the global supply mix (Figure S2). However, the region faces structural challenges. Widespread economic underdevelopment and weak institutional governance increase the risk that resource discoveries may trigger military conflict or political instability, thereby deterring long-term foreign investment.^66^^,^^67^ In addition, artisanal and unregulated mining is common, often associated with severe (environmental, social, and governance) ESG issues such as child labor, hazardous working conditions, and lack of environmental oversight, such as tail leaching management.^68^^,^^69^ These practices not only raise ethical concerns but also limit the marketability of such resources, as many multinational corporations increasing require ESG certification for raw minerals used in their supply chains.^70^^,^^71^ To ensure more effective utilization of AFR’s lithium resources, and to enable local communities to equitably benefit from resource development, there is an urgent need to promote more robust and transparent extraction systems. Achieving this goal will require not only stronger commitment and governance from local governments, but also active collaboration with capable stakeholders, including international investors, development agencies, and responsible industry actors.

The highest cost segment of ore resources is primarily located in the OTH region, including deposits in Russia and Afghanistan. These higher costs are attributed to a combination of low mineral grade, underdeveloped infrastructure, constraints for global trade, fragile political environment and technological limitations.^72^^,^^73^ Especially for Afghanistan, Its mining sector operates under Taliban control, which has long been associated with political instability and international sanctions.^74^ Additionally, data availability and reporting inconsistencies in these regions result in challenges to cost assessments.^75^

#### Global CAC for lithium from unconventional sources

**Figure 5:**
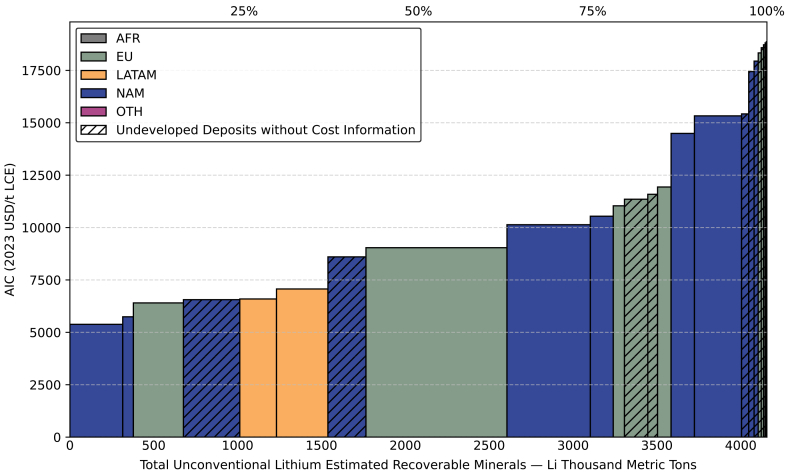
Global lithium CAC from unconventional sources separated by region See also Figures S2C and S5.

According to Figure 5, the ERM lithium from unconventional sources amounts to 4,157 kt Li. Although this volume is minor compared to brine and ore resources, it is increasingly recognized as an important supplementary supply source. Most of these deposits are in EU or NAM, where increasing supply pressures have spurred exploration into unconventional lithium sources. The extraction costs for unconventional sources range from 5,382 to 18,868 USD/t LCE (2023). This suggests that at least a portion of these deposits are economically competitive. Despite cost feasibility, as of 2023, no large-scale unconventional lithium project has reached commercial production (Figure S2).

An important observation is that even for unconventional deposits classified as ore, such as the Zinnwald project in Germany^76^ and the Cinovec project in the Czech Republic,^77^ both based on greisen ore, their output production is not the conventional concentrate. Instead, lithium is refining locally into lithium hydroxide. This may reflect a strategic objective: developed countries invest in unconventional lithium primarily to reduce supply chain risk. To achieve this goal, even if it comes at higher cost compared to processing in lower-cost regions such as CHN, local processing is developed to reduce risk exposure.

The USA holds the largest share of unconventional lithium ERM, with a diverse portfolio of deposit types. The lowest cost project is Rhyolite ridge, a lithium-boron deposit whose AIC is effectively reduced to around 5,000 USD/t LCE (2023) due to the value of co-produced boron. In addition, the US hosts a sizable number of geothermal and oilfield brine projects, such as the Salton Sea project and the Smackover project in Arkansas. These projects are expected to adopt novel direct lithium extraction (DLE) technologies, and their costs fall in the mid-range for unconventional sources, approximately 7000–10,000 USD/t LCE (2023). Importantly, the technological advancements and operational experience gained from these DLE-based projects may help accelerate innovation in conventional brine extraction, reducing environmental impacts associated with traditional evaporation methods.^78^ At the higher end of the cost curve are clay-based deposits, represented by projects in Clayton Valley and Kings Valley. These operations currently exhibit AIC values of around 15,000 USD/t LCE (2023), reflecting technical complexity and lower recovery efficiencies associated with lithium clay extraction.

#### Global lithium CAC for lithium from all sources

**Figure 6:**
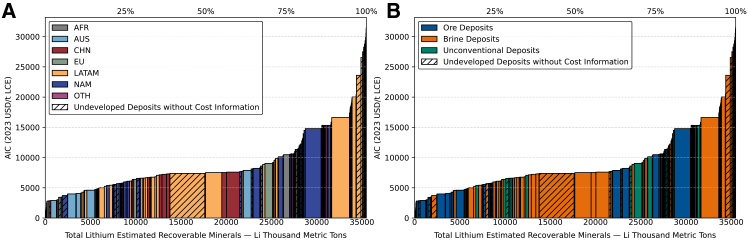
Global lithium CAC from all sources (A) Separated by region and (B) separated by source type. See also Figure S2D, and Table S12.

Figure 6 integrates the data presented in Figures 3, 4, and 5 to construct the global CAC for lithium from all sources. The total ERM is up to 35,493 kt Li with AIC ranging from 1,194 to 31,589 USD/t LCE (2023). It shows that the lowest-cost and already-operating deposits remain concentrated in AUS’s ore mines. In contrast, LATAM brine projects dominate the mid-cost range of the curve. A considerable number of brine deposits, particularly in LATAM and NAM, have competitive cost structures and have already completed prefeasibility studies. Many of them are expected to enter production in the near term, potentially reshaping global supply dynamics and have already contributed to the sharp decline in LCE prices observed in recent years.

Another important observation is that some lithium deposits are projected to have lower production costs than certain currently operating mines, yet they remain undeveloped (Figure S2). A key reason is that the cost estimates in this study reflect purely economic factors, without accounting for potential ESG-related burdens. Consequently, the actual costs of these deposits may be significantly underestimated. As with many forms of mineral extraction, lithium mining, regardless of resource type, is frequently associated with a range of ESG risks, including land degradation, displacement of local communities, excessive water consumption, tailings leaching, and chemical pollution.^78^^,^^79^ Some lithium projects have faced development delays or public protest due to ESG concerns. For example, the Jadar project in Seria was halted following large-scale protests over potential water contamination.^80^ The Rhyolite Ridge project in the United States has faced scrutiny due to its potential impact on a rare plant species, Tiehm’s buckwheat.^81^ As public awareness and regulatory emphasis on ESG issues continue to rise, particularly in economically developed regions, such pressures are expected to intensify. Balancing mineral development with responsible environmental and social governance will thus become an increasingly critical challenge for developers in the years ahead. This study provides an essential foundation by mapping the economic dimensions of lithium resources, serving as a first step toward more integrated assessments that also account for ESG considerations.

### Lithium ERM supply and future demand projections

**Figure 7:**
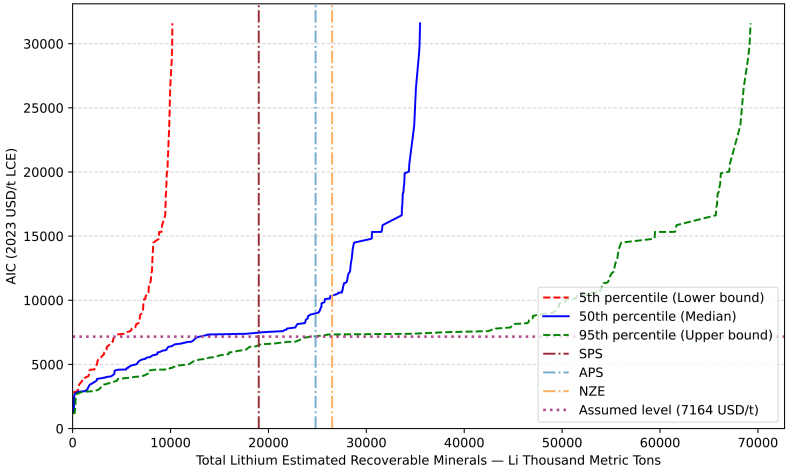
Lithium cumulative availability curve based on different ERM percentile bound and relationship among three IEA scenarios The three curves represent the 5th, 50th, and 95th percentile estimates of estimated recoverable minerals (ERMs), reflecting uncertainty in supply availability. The three vertical lines indicate projected lithium demand under the IEA’s SPS, APS, and NZE scenarios. The horizontal line denotes the upper bound of the May 2025 market acceptable cost estimate for lithium carbonate equivalent (LCE), set at 7,164 (2023 USD/t LCE).

In this study, the ERM values used to construct the CAC are derived through Monte Carlo simulations, to incorporate the uncertainty inherent in the resource estimation process. For the main analysis, the 50th percentile (median) values are used to represent the most likely outcomes. Figure 7 further shows the uncertainty range by presenting the 5^th^ and 95th percentile bounds. Under the 5^th^ percentile scenario, the total global lithium ERM is only 10,189 kt Li, while the 95^th^ percentile reaches as high as 69,253 kt Li, highlighting the substantial uncertainty surrounding long-term lithium availability.

To evaluate whether various supply scenarios can meet future lithium demand and at what cost, three demand scenarios from the IEA are adopted: the Stated Policies Scenario (SPS), the Announced Pledges Scenario (APS), and the Net Zero Emissions by 2050 Scenario (NZE). These scenarios reflect increasingly stringent climate policy commitments. SPS represents a baseline pathway in which countries comply with currently implemented or officially announced policies. APS incorporates all national climate and energy targets that have been publicly declared. NZE outlines a global pathway to net-zero greenhouse gas emissions by 2050.^21^ Each of these scenarios projects a progressively greater reliance on renewable energy and electric vehicles, thereby driving up lithium demand. The IEA has published both the actual demand for lithium in the base year (2023) and projected demand for 2050 under each scenario. Assuming linear growth over the period, total cumulative lithium demand can be estimated (Table 2), and relevant levels also shows in Figure 7.

**Table 2:** Lithium demand based on IEA scenarios estimation (kt Li)

Scenario	SPS	APS	NZE
2023 demand	163	163	163
2050 demand	1,196	1,607	1,728
Total demand	19,062	24,816	26,511

By overlaying the total cumulative lithium demand estimates onto the CAC curve, it is possible to identify the AIC level at which all demand is satisfied. It represents the cost threshold required to ensure sufficient lithium supply under each scenario. The resulting AIC level under different demand scenarios and supply bounds are summarized in Table 3.

**Table 3:** Comparison of AIC levels under different IEA scenarios by ERM percentile boun**d**

	SPS	APS	NZE
5^th^	NaN	NaN	NaN
50^th^	7,481	8,980	10,368
95^th^	6,514	7,197	7,334
Rise (95^th^ to 50^th^)	15%	25%	41%

NaN means insufficient; rise (95th to 50th) means the AIC level increased percentages from 95th percentile bound to 50th percentile bound under different IEA scenarios.

The results show that under the 5^th^ percentile supply scenario, lithium requirements under all three IEA climate scenarios cannot be met, indicating a potential risk of severe undersupply in the most conservative case. Conversely, under the 50^th^ and 95^th^ percentile supply scenarios, the available ERM is sufficient to meet lithium demand across all three IEA scenarios. However, a notable difference lies in the variation of the AIC level. As shown in Tables 2 and 3, a horizontal comparison across scenarios reveals that pathways with higher climate ambition are associated with higher lithium demand, which in turn requires the exploitation of higher-cost deposits, leading to a higher AIC level under both supply bounds. A vertical comparison between 50^th^ and 95^th^ percentile bounds further shows that, under the more conservative 50^th^ percentile supply assumption, the AIC level required to meet each climate scenario is consistently higher. The increase ranges from 15% to 41%, with a more pronounced rise observed under the more stringent decarbonization scenarios, indicating that tighter supply assumptions amplify the cost pressure particularly in high-demand pathways.

A recent market price of lithium carbonate is introduced as a reference point for comparing the projected AIC level. While lithium product prices are highly volatile and the relationship between market price and AIC level cannot be reliably established from a long-term perspective, such a comparison still offers useful insight. As of May 2025, the Shanghai metal market reported a market price in USD 8,597/t LCE (in 2023 USD; USD 8,941 in nominal 2025 terms) for battery-grade lithium carbonate.^82^ Assuming a 20% profit margin, the maximum acceptable AIC would be 7,164 USD/t LCE (2023). At this price level, the 5th, 50th, and 95th percentiles correspond to AIC levels of 4,284, 12,974, and 24,387 kt Li, respectively. A comparison of these figures with the lithium demand projections under the three IEA scenarios is presented in Table 4, indicating supply coverage ranging from merely 16% to as high as 128%. Notably, it is only under the 95th percentile supply scenario, coupled with the SPS demand projection, that the primary supply sources can hit anticipate demand at current market cost. Although this comparison is approximate, omitting the effects of long-term price fluctuations, and feedback effects between price and extraction costs, it nonetheless highlights the mounting cost pressure on future lithium supply. This also implies that under such conditions, meeting demand will require either higher lithium prices or increase reliance on recycling, particularly if breakthroughs allow recycling costs to fall closer to those of primary extraction.

**Table 4:** Comparison of lithium supply at or below current AIC level in 5th, 50th, and 95th percentile with IEA demand scenarios

	SPS	APS	NZE
5^th^	22%	17%	16%
50^th^	68%	52%	49%
95^th^	128%	98%	92%

The current AIC level is estimated as 7,164 (2023 USD/t LCE), at this AIC level, 5th, 50th, and 95th percentile available amounts LCE are 4,284, 12,974, and 24,387 kt Li. The cumulative lithium demand by 2050 based on IEA scenarios are 19,062 kt Li (SPS), 24,816 kt Li (APS), and 26,511 kt Li (NZE).

**Table 5:** Triangular distribution data for calculating coefficient A: LATAM as a sample

	Aggregated USGS	Aggregated min	Aggregated mean	Aggregated max
Estimated Resources (Li content Mt)	59.57	46.99	60.30	75.25
Normalized percentage	99%	78%	100%	125%
Parameter	–	a	b	c

### Sensitivity analysis for global lithium CAC from all sources

In the section “lithium ERM supply and future demand projections,” Monte Carlo results show uncertainty propagating through each stage of CAC construction and accumulating, ultimately producing a sizable spread in outcomes. To clarify the contribution of individual parameters, and thereby enhance transparency and reliability, a sensitivity analysis is performed. This enables a focus on the most consequential uncertainties. Before testing, parameters are grouped into two sets: those affecting ERMs and those affecting the AIC. Detailed definitions, baseline values, distributional assumptions, and links to the uncertainty framework are provided in Methods, “parameters summaries and sensitivity analysis setting.”

**Figure 8:**
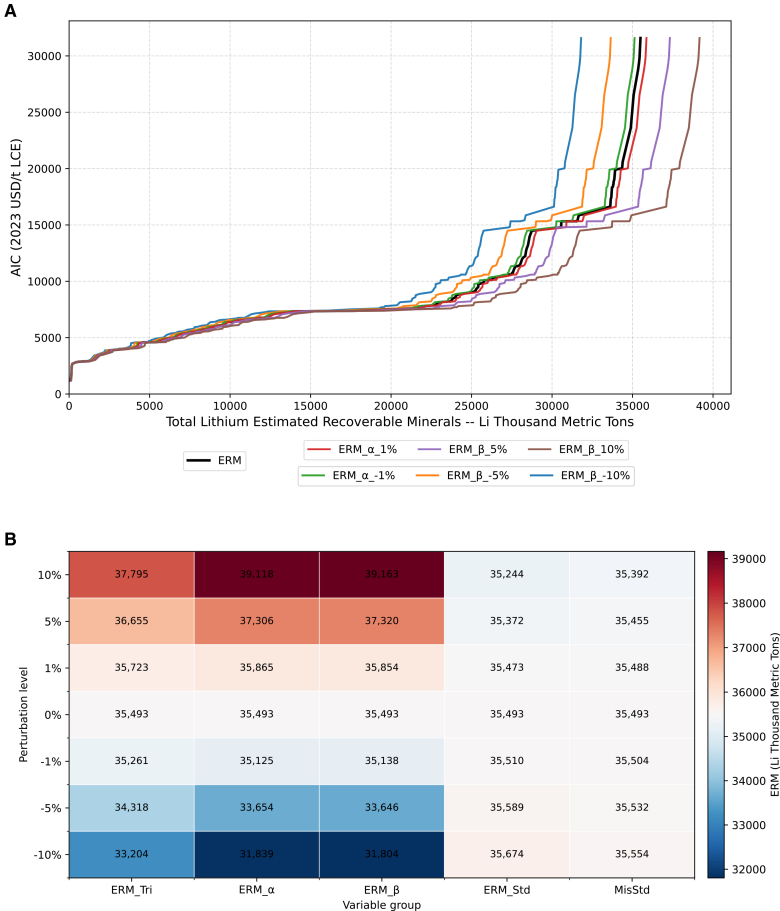
Lithium CAC sensitivity analysis results for ERM-related variables (A) CAC results, including sensitivity analysis results, only shows baseline curve and biggest change variable curves for each perturbation level (±1%, ±5%, ±10%). (B) Heatmap showing the influence of each variable group on ERM across perturbation levels.

According to Figure 8A, the overall CAC is notably sensitive to parameter variations, with the degree of deviation increasing markedly when the ERM figure over 20,000 and AIC figure over 7,500. This pattern likely reflects the difference in data quality along the curve: deposits in the lower-cost range are typically in operation and well-studied, whereas those in the high-cost tail are less explored due to lower grades, remote locations, higher impurity levels, and limited supporting infrastructure. These factors lead to data scarcity and thus greater uncertainty. Another contributing factor is that uncertainty accumulates throughout CAC construction, amplifying deviations in the tail section.

As shown in Figure 8B, under a ±1% perturbation, factor *α* exhibits the largest impact forcing total ERM figure move from 35,493 to 35,865 (+1.048%) and 35, 125 (−1.037%), followed closely by factor *β*. At the ±5% and ±10% perturbation levels, factor *β* becomes the dominant driver, with the total ERM values reaching 37,320 (+5.147%) and 33, 646 (−5.204%), and 39,163 (+10.34%) and 31,904 (−10.11%) respectively. The dominance of these two factors indicates that estimates of physical (reserves level volumetric), and technical uncertainty exert the greatest influence on the total ERM. This impact is particularly pronounced for deposits located in the tail-end of the curve, where data scarcity and geological complexity amplify uncertainty. Variations in the upper and lower bounds of the triangular distribution for coefficient A, which primarily reflects physical uncertainty (resource level volumetric), show relatively stable outcomes (37,795 [+6.486%] and 33,204 [−6.449%]) at the 10% level. The variables ERM_Std (the standard deviations of factor *α* and factor *β*) and MisStd (the standard deviations of factor *α* and factor *β* adjustment applied when cost information is missing) also contribute to overall variability, though their influence remains inconspicuous compared with the baseline parameter shifts.

**Figure 9:**
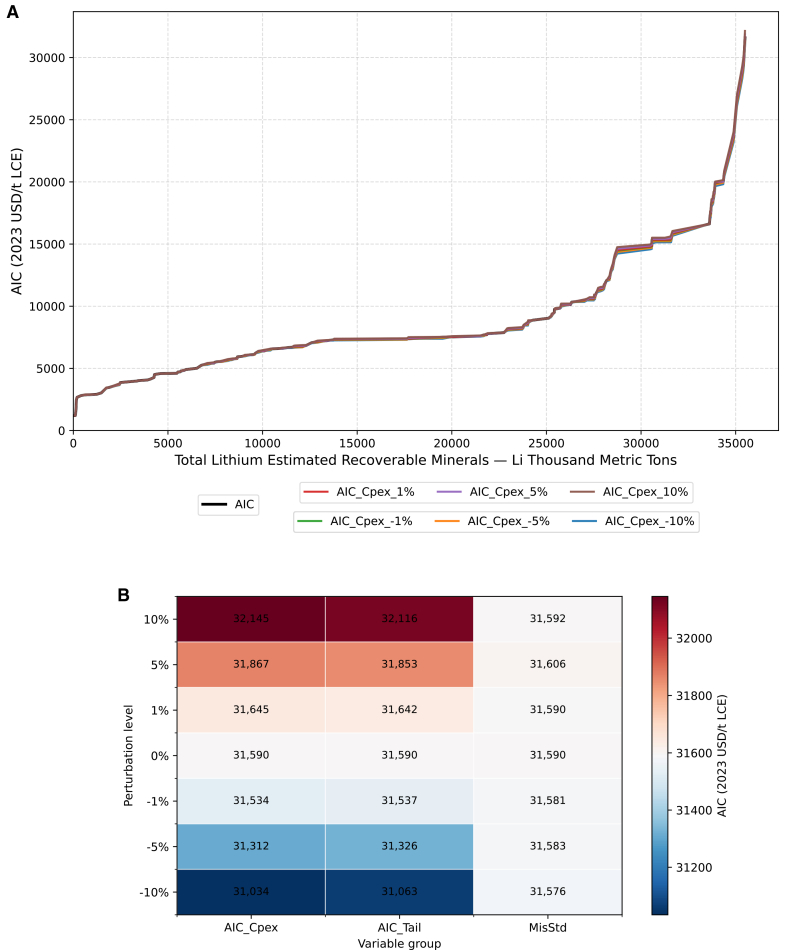
Lithium CAC sensitivity analysis results for AIC-related variables (A) CAC results including sensitivity analysis results, only shows baseline curve and biggest change variable curves for each perturbation level (±1%, ±5%,±10%). (B) Heatmap showing the influence of each variable group on AIC across perturbation levels.

Regarding the impact of uncertainty on AIC, Figure 9A presents a contrasting picture. Variations in the economic variable groups have only a limited effect on the overall CAC, with resulting curves remaining highly concentrated. This indicates that cost-related uncertainties exert a relatively minor influence on the aggregate outcomes. A likely explanation is that the parameter AIC_Cpex (used to estimate initial capital cost, ICAPEX) accounts for only a small proportion of total project costs and applies solely to undeveloped deposits lacking explicit ICAPEX data. Similarly, AIC_Tail (the tail-uplift coefficient applied when inferring AISC for deposits without cost data) functions merely as a minor adjustment to undeveloped deposits without production cost information. MisStd, as a factor both influence AIC and ERM, also represents only a small refinement to the underlying distribution. Compared with data-rich depletion curve, these factors serve mainly as secondary adjustments. Overall, this suggests that uncertainty affecting CAC costs is more likely to stem from terminology uncertainty, as it determines which definitions are adopted when constructing the depletion curve and, ultimately, the CAC itself.

Figure 9B provides a quantitative view of these relationships. Within the AIC-group, AIC_Cpex consistently shows the largest influence across all perturbation levels; at the ±10% level, the maximum AIC varies from 51,590 to 32,145 (+1.757%) and 31,034 (−1.760%). The next most influential factor is AIC_Tail, with AIC values of 32,116 (+1.665%) and 31,034 (−1.668%) at the same perturbation level. The smallest contribution comes from MisStd; as it modifies the underlying distribution rather than shifting central estimates, a wider spread does not necessarily translate into a larger effect on final AIC values (for example +5% leads to 31,606, but +10% 31,592).

### Future research direction: Uncertainty related to CAC application

In the introduction, uncertainties affecting lithium availability are classified into three categories, among which those related to CAC application reflect broader systemic influences, including ESG risks, advances in recycling technologies, geopolitical constraints, and unforeseen future shocks. Following Walker et al. (2003),^50^ this type of uncertainty arises from features of the external environment and their impacts on the system, which makes statistical characterization challenging. Consequently, scenario analysis offers a more suitable approach for representing and exploring such uncertainties. As these factors cannot be meaningfully incorporated into a baseline CAC, attempting to embed them within the curve would add complexity, create confusion, and ultimately fail to improve its accuracy.^83^^,^^84^

A more effective treatment is to use the CAC as a reference framework for scenario design. Consistent with the study’s forward-looking agenda, CAC outputs can serve as inputs to IAMs, where regional and resource-type-specific stepwise supply blocks define quantities and costs. This structure allows for the representation of cross-regional lithium trade and the exploration of geopolitical risks, while downstream value-chain components are integrated through scenario assumptions. In this way, IAMs can investigate how uncertainties in lithium availability influence pathways toward decarbonization under alternative futures. A simplified morphological scenario framework is presented in supplemental information section 4 including Table S15 and Figure S9, illustrating how CAC-derived data can underpin scenario construction and how it interfaces with other uncertainties driven by external factors shaping the lithium value chain.

### Conclusions

This study develops a bottom-up database including ERMs and AICs for lithium deposits. Using this database, a CAC is constructed to assess long-term supply potential. Data for a total of 188 deposits, including brine, ore, and unconventional sources, are collected and analyzed. To address inconsistencies across data sources, resource definitions were first clarified and organized into a tiered classification to guide data selection. Reported values were then integrated using triangular distributions and combined with normal distributions to derive probabilistic ERM estimates. For production costs, diverse metrics were harmonized by applying conversion formulas that aligned different cost components and reporting conventions into a common structure. The resulting ERM totals 35,493 kt Li, with AIC ranging from 1,194 to 31,590 USD/t LCE (2023). Brine sources concentrate on middle and highest range, ore dominated low-cost supply, and unconventional sources show long-term potential despite limited current activity. Besides, regional levels of economic development and potential ESG-related risks also play a vital role in shaping the feasibility and progression of mineral operating activities.

Monte Carlo simulation provides uncertainty bounds, which, when combined with IEA demand scenarios, highlights increasing cost pressures under stricter climate pathways and more conservative resource assumptions. While the physical potential of lithium is not a constraint, converting resources into reserves in an economically and socially viable way remains challenging. The sensitivity analysis further evaluates how different sources of uncertainty affect CAC construction under varying perturbation levels. Overall, physical and technical uncertainties exert the greatest influence on the estimation of ERM, while terminology uncertainty plays a more decisive role in shaping the AIC, and thus the cost dimension of the CAC.

### Limitations of the study

This study comes with a set of limitations and opportunities for further work.

From terminology perspective, the construction of the CAC framework is challenged by substantial inconsistencies in terminology across data sources. Despite the clarification and harmonization efforts undertaken in this study, it still faced with difficulties for data integration. Enhanced international cooperation and the development of standardized reporting practices would help to alleviate these terminology-related uncertainties in future research. From a methodological perspective, it applies a depletion curve approach to estimate unknown cost data. This essentially assumes that deposits with known costs within a region share similar depletion characteristics with those for which cost data are unavailable. Although this is a relatively coarse estimate, it represents a careful effort given the current level of data availability. The precision of these estimations is expected to improve considerably as more data on individual deposits become available or can be reliably forecast in the future. Regarding data utilization, ICAPEX and royalty costs have often been represented by regional or deposit-type averages due to limited data sources. A more scientific assessment of these components would allow for more detailed and robust cost predictions. In addition, the model does not yet account for the uncertainty related to CAC application.

## Resource availability

### Lead contact

Further information and requests for resources and materials should be directed to and will be fulfilled by the lead contact, Dongping zhou (dongping.zhou.20@ucl.ac.uk).

### Materials availability

This study did not generate new materials.

### Data and code availability

• The data used in this study can be found in supplemental information and https://doi.org/10.5281/zenodo.17847951.
• The code used in this study for analysis and visualization can be found at: https://doi.org/10.5281/zenodo.17847951.
• Any additional information required to reanalyze the data reported in this article is available from the lead contact upon request.

## Acknowledgments

The author gratefully acknowledges the University College London (UCL) for providing the academic environment and research training that made this work possible.

The open access publication of this article was supported through the 10.13039/501100000821JISC agreement between UCL and the publisher, which covered the article processing charge.

## Author contributions

D.Z. conceived the study, designed the research framework, conducted the data collection and analysis, developed the modeling approach, interpreted the results, and wrote the original draft of the manuscript. S.P., B.V., and P.D. provided academic supervision throughout the research process, reviewed the manuscript, and offered critical comments that helped improve the clarity and quality of the work. S.P. made substantial contributions to the revision and refinement of the manuscript. All authors reviewed and approved the final version of the manuscript.

## Declaration of interests

The authors declare no competing interests.

## STAR★Methods

### Key resources table

**Table:** 

REAGENT or RESOURCE	SOURCE	IDENTIFIER
**Deposited data**		

Lithium deposits data	Reports, Datasets, and literature review	https://doi.org/10.5281/zenodo.17847951

**Software and algorithms**		

Python	This Paper	https://doi.org/10.5281/zenodo.17847951
IBM SPSS Statistics 23	https://www.ibm.com	N/A

### Method details

#### Lithium resource data clarification

This section presents the data foundations for constructing the Cumulative Availability Curve (CAC). It introduces the classification of lithium resource types, clarifies key terminologies and their interrelationships, and summarises the data sources on which the analysis is based.

#### Types of lithium resources

Lithium is among the top 30 most abundant element in the Earth’s crust,^85^ occurring in a variety of geological settings, with distinct modes of occurrence and extraction methods. To enable a more accurate assessment of production costs at the source location stage of the value chain, this study categories lithium sources into three principal types: pegmatite-type ores (Ore), Salt Lake brines (Brine), and other unconventional deposits. Lithium content is used as a normalisation metric.

##### Lithium from ore

Pegmatite-type ores represent the principal ore sources of lithium, with spodumene as the dominant mineral, accompanied by lesser amount of lepidolite and petalite. Their importance arises from typically high lithium grades, straightforward mineralogy, and well-established beneficiation processes.^40^^,^^86^^,^^87^ Pegmatites typically form in the late stages of magmatic evolution, when lithium as an “incompatible element”, accumulates in the residual melt and becomes concentrated in these rocks.^88^ Yaksic and Tilton^43^ estimate that around 50% of lithium contained in pegmatites deposit resources are recoverable. Australia dominants global lithium ore resources, holding 30%–40% of the world’s total, largely in high-grade spodumene supported by advanced mining infrastructure.^89^ Significant deposits also exist in Quebec, Canada, and Sichuan and Jiangxi provinces in China.^90^ Once mined, lithium ore is processed through beneficiation to concentrate, involving crushing, grinding, gravity separation, and flotation.^91^ These steps typically yield a concentrate containing approximately 6% lithium oxide and then be sold.

##### Lithium from brine

Salt Lake lithium brines form through crustal extension that creates closed basins, leaching of lithium from bedrock by geothermal fluids, and evaporation under arid conditions.^92^ It is estimated that approximately 45% of the lithium in brines is recoverable.^43^ Globally, lithium brines are spatially clustered in South America’s “Lithium Triangle” (Chile, Argentina, and Bolivia) and in western China, areas that remain less developed in economic and technological terms.^93^ Unlike hard-rock ores, lithium brines are usually processed directly into lithium compounds through evaporation method. Recently, Direct Lithium Extraction (DLE) has emerged as a more efficient and environmentally friendly alternative.^94^ Compared to ore-based lithium projects, brine developments have lagged due to higher capital needs, sensitivity to brine chemistry, and Environmental, Social, and Governance (ESG) concerns.^78^

##### Lithium from unconventional sources

The growing demand of lithium has intensified attention on diversifying beyond ore and brine sources, despite the economic and technological challenges many of them still face.^95^ Collectively referred to as unconventional lithium sources, these deposits are typically characterised by lower concentrations or more complex extraction processes. They can be classified into three categories: unconventional brine,^95^^,^^96^^,^^97^ unconventional ore^98^ and clay,^99^^,^^100^^,^^101^ each encompassing several subtypes. Like brines, these unconventional lithium sources are generally processed straight into lithium compounds, by passing a concentrate stage, for subsequent sale and transport. Some other potential lithium sources, such as seawater and the upper continental crust, have been explored in studies^102^^,^^103^; however, they are excluded from the scope of this study due to the absence of foreseeable plans for their commercial development.

#### Terminology clarification

Give the diverse and inconsistent use of terminology across lithium data sources, this section aims to standardise key definitions and delineate their interrelationships to enable meaningful cross-comparison. This can significantly mitigate the impact of terminology uncertainty when constructing lithium database and Cumulative Availability Curve (CAC).

##### Resources and reserves

Classifying the terminology of mineral resources and reserves is important for mineral industry, with ongoing attempts to clarify these different definitions.^104^^,^^105^

Two major frameworks are commonly used to classify and report mineral resources and reserves: the CRIRSCO framework (Committee for Mineral Reserves International Reporting Standards)^106^ and the McKelvey^107^ framework. Both rely on two key dimensions, geological confidence and modifying factors, to distinguish between mineral resources, reserves, and their respective subcategories. In general, resources refer to mineral concentrations with current or potential economic value, whereas reserves represent portions that are currently economically extractable. The McKelvey framework places a stronger emphasis on economic feasibility, while CRIRSCO expands the definition of modifying factors to include legal, environmental, and social considerations. Besides, CRIRSCO is typically used for project-level reporting by companies, whereas McKelvey is applied in regional or national assessments of mineral potential, particularly by institutions such as the USGS.

Additionally, some data sources do not clearly define their categories, leading to significant uncertainty when comparing lithium resource estimates. In this study, resources were chosen due to the wide availability across datasets. Conversion factors were then applied to estimate reserves. Where possible, “Measured and Indicated (M&I) Resources” were prioritised, as they are accepted as meeting criteria for reserve conversion. In their absence, broader classifications such as total resources, ultimate recoverable resources (URR), or resources were used.

To bridge geological availability with practical supply, this study introduces the working concept of Estimated Recoverable Minerals (ERM). ERM represents the portion of resources realistically extractable and refinable under current conditions, accounting for extraction efficiencies and processing losses. This provides a more actionable measure of the quantity usable for industrial and societal needs. The detailed information of ERM can be found in Section (six-step method for building a cumulative availability curve (CAC)).

##### Production cost

Numerous cost categories are used in the mining industry and appear across databases and reports, including Total Cash Cost (TCC),^54^ Capital Cost (CAPEX) and Operating Cost (OPEX),^108^ Operating cost including by-product credits,^109^ C1 cash cost,^110^ Initial Capital cost (ICAPEX) and Sustaining Capital cost (SCAPEX),^111^ All-in-Sustaining Cost (AISC),^112^ All-in Cost (AIC),^113^ and Royalty cost.^114^ Some sources also use general terms like “production cost”. These inconsistencies complicate cross-source comparisons and introduce data uncertainty.

This study adopts AIC as the standard metric to capture the full cost of lithium extraction and processing. To ensure clarity and consistency, the relationships among these cost concepts are outlined below.

AIC represents the total cost of producing lithium, incorporating OPEX, SCAPEX, and for undeveloped deposits, ICAPEX. For operating mines, ICAPEX is excluded as it is a sunk cost. Thus, AIC better indicates forward-looking production costs. The calculation formula for the AIC is presented in Section (six-step method for building a cumulative availability curve (CAC)) Equation 4. Besides, to enable a consistent cost comparison between operating and undeveloped deposits, the concept of All-in-Sustaining Cost (AISC) is introduced. AISC includes OPEX and SCAPEX, capturing the recurring costs to sustain production. According to IFRS,^115^ OPEX comprises direct production expenses such as materials, labor, and overhead, while SCAPEX covers future maintenance or expansion needs once operations commence. The detailed information about AISC and calculation formula is presented in Section (six-step method for building a cumulative availability curve (CAC)) Equation 3.

Operating costs are reported under varied terms. This study prioritises “operating costs including by-product credits”, where costs are offset by revenues from by-products such as sodium hydroxide (NaOH), offering the clearest view of standalone lithium costs. If unavailable, general OPEX or C1 cash costs (focuses on the direct production cost)^116^ are used, followed by adjusted TCC. Per the S&P,^117^ TCC includes labor, energy, reagents, other onsite, offsite, shipping, and royalties. To focus on site-level extraction and processing, offsite and shipping costs are excluded, with the adjusted TCC serving as a proxy for AISC. Royalty costs, payments based on production value or volume, vary widely across sources. Although royalties are policy-sensitive and may fluctuate, they are included in AIC here to provide a more realistic view of project costs and facilitate cross-country comparisons.

#### Source of data

To explore long-term lithium availability, this study constructs a Cumulative Availability Curve (CAC), drawing on three main data sources: global and national datasets from official organisations, academic literature, and company reports. These collectively support mapping the distribution of lithium resources alongside their production costs.

##### Governmental and other public and commercial organisation datasets

Country-level statistics are key sources for lithium estimates, with the USGS Mineral Commodity Summaries (MCS) among the most cited. This annual report offers data on global lithium production, resources, reserves, and sectoral use, and its consistency and open access have made it foundational for academic work.^118^^,^^119^ However, critiques highlight its limited methodological transparency, absence of US data, and possible underestimation due to non-reported private Figures.^120^^,^^121^ Reports from other governments, such as the British,^122^ Argentina,^123^ and Australia,^124^ serve as important complements.

Organisational datasets include open-access sources like British Petroleum^125^ and Energy Institute,^126^ which occasionally report country-level reserves, and commercial providers such as S&P, Wood Mackenzie, and Benchmark Mineral Intelligence. The high cost of licensed data limits academic use.^127^^,^^128^ Representative sources in this category include S&P,^54^ Wood Mackenzie, and Benchmark Mineral Intelligence. This study primarily relies on S&P’s Mine Economics database, covering over 90% of global lithium production with detailed cost structures. Data from Wood Mackenzie and Benchmark are included indirectly via secondary literature.^58^^,^^112^

##### Academic literature

Academic studies increasingly explore lithium availability, though supply-side analyses are fewer due to data constraints. For production costs, Yaksic and Tilton^43^ compiled figures from a variety of industry sources, Ambrose and Kendall^58^ estimated average cost across different grade-types categories, incorporating standard deviations to capture uncertainty, and Fleming et al.^41^ employed a multiple linear regression model using variables grade and location to predict production costs. Only Fleming et al.^41^ explicitly defined production cost as operating plus capital expenditures, excluding royalties and taxes.

Resource estimation studies are more extensive.^16^^,^^36^^,^^39^^,^^40^^,^^43^^,^^58^^,^^129^^,^^130^^,^^131^ However, there are substantial discrepancies among their results. On the one hand, these differences can be attributed to variations in data sources or statistical methodologies, indicating a considerable degree of underlying uncertainty. On the other hand, inconsistencies may also stem from translation-related naming issues, which introduce additional ambiguity into the analysis. For example, Mohr et al.^16^ refer to “Jiajika” and “Giajika” as separate deposits, although they represent the same deposit in Chinese. Similarly, Fleming et al.^41^ list “Dongtai Jinaier Salt Lake” and “East Taijinar lake”, as well as “Qarhan Salt lake” and “Chaerhan Salt Lake (Qaidam)”, each pair denoting the same deposit but treated as distinct due to translation differences. In this study, these factors have been carefully considered and cross-checked to ensure the robustness and reliability of the findings.

##### Company report

Company disclosures, including financial statements and prefeasibility studies, are crucial for deposit-level data. Prepared to meet listing standards, most follow CRIRSCO principles (details in terminology clarification). Prefeasibility reports are especially valuable, offering detailed insights into resources, costs, and early production planning.

#### Data preprocessing and database construction

The database comprises two classes of data: (i) relatively certain attributes (deposit location, type, operating status); and (ii) uncertain attributes, specifically “resources and reserves” and “production cost”. For the former, confirmation is made via the source mentioned in the section (source of data). For the latter, a consistent harmonisation protocol is applied: quantities are converted to common units (Mt lithium content; 2023 USD/t LCE) (Tables S1 and S2), provenance and reporting standards are recorded, and any conversion rules are documented. For “resources and reserves”, owing to heterogeneous sources and the absence of trustworthy conversion factors, “Resources” as represented terminology has been selected via a ranking procedure based on definitional accuracy. The ranking logic is explained in the “resources and reserves” subsection of “terminology clarification”. The minimum, maximum and mean across available entries are then computed to support subsequent uncertainty assessment. For the production cost data, Terminology clarification specifies that the target metric is the all-in cost, comprising All-in Sustaining Cost (AISC) and initial capital cost (ICAPEX). These figures are obtained either directly from reported sources or by calculation. The calculations require, annual production, life-of-mine (LoM), total initial capital cost (ICAPEX), total sustaining capital cost, and operating cost (OPEX); the approach to deriving OPEX is detailed in the production cost subsection of “terminology clarification”. Once these values are obtained, the foundational database is assembled.

It should be noted that selection among alternative definitions is made only within the same source: if a source reports resources or production costs under multiple terminologies, the more accurate terminology is adopted based on the ranking. By contrast, across different sources, despite substantial effort to map relationships, there is no scientifically defensible conversion factor that would allow horizontal comparison; imposing an ad hoc factor would risk amplifying uncertainty.^132^^,^^133^ Accordingly, data from different sources are collated, units are standardised, and means, minima and maxima are reported to characterise ranges and central tendency; subsequent analyses then assess the associated uncertainty using distributions, Monte Carlo analysis, and sensitivity analysis. Database detailed information provided in (Tables S3–S8).

#### Six-step method for building a cumulative availability curve (CAC)

To quantitatively assess future lithium supply under varying cost levels, this study develops a Cumulative Availability Curve (CAC) through a structured 6-step methodology (Figure S1). The main challenges involve estimating production costs for data-limited deposits and managing uncertainties in resource and cost figures.

First, data are collected and organised by geographic region and resource type. Second, a Monte Carlo simulation estimates the Estimated Recoverable Minerals (ERM) for each deposit. Third, for deposits with cost data, the All-in Sustaining Cost (AISC) is calculated and combined with ERM to build regional depletion curves. In the fourth step, these curves are used to impute costs for deposits lacking direct estimates. Fifth, undeveloped deposits incorporate ICAPEX, resulting in an All-in Cost (AIC) that captures total project expenditure. Finally, in the sixth step, regional CACs by resource type and area are aggregated into a global CAC.

##### Step 1

Lithium deposit resource and production cost data from various sources are consolidated and first categorised by source type. These deposit and country-based data are then aggregated by region to do further analysis. In this step, 12 distinct analytical groups are created (Tables S9 and S10).

##### Step 2

The second step estimates the Estimated Recoverable Minerals (ERM) for each deposit, representing the amount of lithium expected to be recovered as production output. This is calculated using Equation 1:

(Equation 1) ERM=ER∗CoefficientA∗α∗β

Where *ER* represents the average figure of Estimated Resources from different data sources; *Coefficient A* is an adjustment factor that accounts for uncertainty in resource estimation at the deposit level; *α* represents the conversion ratio from resources to reserves; *β* denotes the recovery efficiency during the extraction and refining processes.

The calculation of *Coefficient A* is based on a triangular distribution, which is chosen for its simplicity and flexibility in handling datasets that are skewed and where limited information is available to the actual shape of the underlying distribution.^45^^,^^134^ The associated probability density function is shown in Equation 2:

(Equation 2) $$f\left(x|a,b,c\right)=\left\{ \begin{array}{cc} \frac{2\left(x-a\right)}{\left(c-a\right)\left(b-a\right)}; & a\leq x\leq b\\ \frac{2\left(c-x\right)}{\left(c-a\right)\left(c-b\right)}; & b<x\leq c\\ 0; & x<a,x>c \end{array}\right.$$

Where *a* is minimum parameter; *b* represents model parameter (can be the mode, mean, or median); *c* is maximum parameter; *x* denotes population sample from the distribution.

In this study, triangular distributions are constructed at the regional level, with parameters *a*, *b*, and *c* tailored to each region to reflect local uncertainty. For each lithium deposit, minimum, mean, and maximum estimate resources are assigned based on various data sources. These are then aggregated to derive regional-level minimum, means, and maximum, which are subsequently compared with USGS top-down estimates.

To construct the triangular distribution, the aggregated mean is adopted as the benchmark value and set as parameter *b*, normalized to 100%. Then, four values are expressed as percentages relative to the aggregated mean. The lowest of the four is assigned as parameter *a* (minimum of the distribution), and the highest as parameter *c* (maximum of the distribution). This method allows for region-specific representations of uncertainty while ensuring consistency across resource assessments. An example for LATAM is presented in Table 5.

The parameter *α* represents the adjustment coefficient that converts resource estimates into reserves, capturing the transition from potential economic value to actual economic value. Based on the projections by Yaksic and Tilton,^43^ this study adopts a baseline value of 50% for ore and 45% for brine and unconventional sources. The parameter *β* represents the conversion efficiency of lithium content from the feed-in stage to final output. For ore-based deposits, a baseline value of 85% is applied, accounting for material losses during extraction, crushing, and milling related processes. For brine and unconventional sources, a baseline of 59% is used to reflect losses during extraction and refining. The parameter *α* and *β* are further adjusted based on regional information (Table S11). A normal distribution with a standard deviation of 20% for *α* and 10% for *β* is applied to capture uncertainty, reflecting the assumption that extreme high or low values are less likely to occur.^45^ Furthermore, for deposits lacking production coarse information, their standard deviation values shall be increased by 5% to reflect the heightened uncertainty arising from their more incomplete data.

After obtaining these three parameters, a Monte Carlo simulation is conducted with 1000 iterations to generate a distribution of ERM values for each deposit.

##### Step 3

The third step aims to construct regional depletion curves using deposits with existing cost information, combined with the ERM values derived in step 2. A regional depletion curve illustrates the relationship between cumulative recoverable resources and their associated production costs within a specific region. This approach has been applied in resource cost assessments,^44^^,^^45^ and in this study, serves as the basis for estimating the production cost of deposits without direct cost information.

the key challenge in this step lies in harmonizing production cost data reported across various sources. To ensure comparability between operating mines and undeveloped deposits, All-in-Sustaining Cost (AISC) is selected as the standard cost metric, as it excludes initial capital expenditure and thus allows all deposits to be assessed on a consistent basis. The detailed calculation of AISC is presented in Equation 3:

(Equation 3) AISC=(OPEX(includingRoyalty)+(TotalSCAPEX/(LOM∗AP)))∗R_t

Where *LOM* is Life of Mine; *AP* is annual production (LCE); *R*_*t*_ represents the time adjustment factor, introduced to account for temporal cost differences. Since all costs are expressed in constant 2023 USD, values from different years are adjusted to real 2023 terms using the US inflation rate^135^ (Table S2).

By combining the AISC with the corresponding ERM and normalizing the ERM to express it as a percentage, a resource type, regionally based cost depletion curve can be constructed to reflect the overall extraction cost structure within each group. The results can be found in (Figures S3–S5).

##### Step 4

The objective of this step is to estimate the production cost of deposits lacking AISC information by leveraging those deposits with AISC costs. While a straightforward approach would involve using linear regression models with appropriate predictors, prior studies have shown that variables such as resource size, grade, evaporation rate, and the Mg/Li ratio may influence lithium production costs.^34^^,^^136^ However, correlation analysis conducted in this study revealed no strong and consistent relationships between grade and AISC (Tables S13 and S14; Figure S8). Cost depletion curve-based imputation methods are adopted for this study, which integrate geological scarcity and cost-effectiveness logic into a coherent framework.^45^^,^^137^ Specifically, deposits without cost data are ranked in descending order by resource size, reflecting the principle of economies of scale, and then aligned to the constructed cost depletion curve to ensure internal consistency in regional cost distribution. Besides, to reflect potential underestimation of costs in the high-cost tail due to declining grades, a tail-risk adjust factor is applied. For deposits lacking explicit cost data, AISC is uprated by 10% for entries falling within the 50–80% cost quantile of the CAC, and by 20% for those within the 80%–100% quantile. This heuristic adjustment acknowledges grade-related cost escalation where evidence is sparse, while keeping the baseline conservative.

##### Step 5

The fifth step incorporates ICAPEX to reflect the construction cost of undeveloped mines in future production planning. Operating mines are identified using the S&P data for 2023, and for these deposits, AIC is assumed to equal AISC. For undeveloped mines, the AIC is calculated either as the sum of AISC and a project specific ICAPEX, or by applying a type-level average multiplier to the AISC. The calculation of this step shows in Equation 4:

(Equation 4) $$AIC=\left\{ \begin{array}{cc} AISC; & operating\;mines\\ AISC+ICAPEX; & undeveloped\;deposit\;with\;ICAPEX\\ AISC*\left(1+\mu \right); & undeveloped\;deposit\;without\;ICAPEX \end{array}\right.$$

Where *μ* is type-level average percentage of initial capital cost to AISC; *ICAPEX* is Initial Capital cost with formulation shows in Equation 5:

(Equation 5) ICAPEX=(TotalICAPEX/(LOM∗AP))∗R_t

Following this step, type-regional level CACs can be constructed by combining the AIC with corresponding ERM for each deposit (Figures S6 and S7; Figure 5) and relevant data shows in Table S12.

##### Step 6

In the last step, data from all regions and types are aggregated and re-ranked to construct a global CAC. Based on the Monte Carlo simulation results, the global CAC is further characterised by its 5th and 95th percentile bounds, providing an estimate of uncertainty and variability in long-term lithium supply costs. These bounds can be used to compared with different demand scenarios to evaluate the overall uncertainties.

#### Parameters summaries and sensitivity analysis setting

As outlined in the introduction, uncertainty pervades lithium availability estimation. Terminology uncertainty is characterised in terminology clarification and carried through to data preprocessing and database construction. By contrast, uncertainties related to CAC construction are embodied in the parameter choices made when building the curve. To enhance methodological transparency, the principal parameters are summarised in this section.

The first uncertainty arises from the volumetric perspective under the physical dimension, reflecting how much lithium is contained within deposits. In this study, this uncertainty operates at two levels: the resources level and the reserves level. At the resource level, Step 2 of the “six-step method for building a cumulative availability curve (CAC)” computes the ERM using *Coefficient A*, which captures the variability of regional resource totals across different data sources. For each region, *Coefficient A* follows a triangular distribution calibrated to the observed range in the database. At the reserves level, additional uncertainty arises from overlaps with terminology uncertainty, as the conversion from resources to reserves involves “modifying factors” that determine which portion of the volumetric estimate is economically extracted at the time of determination.^55^ These aspects are discussed in the section (terminology clarification). The associated uncertainty is represented through the resource to reserves conversion within the ERM formulation via the *α* factor. The baseline figure 50% and 45% for ore and brine were set in the Step 2.^43^ For Unconventional sources, the baseline is aligned with brine, reflecting data scarcity and substantive similarities in processing, such as processing paradigm,^138^ reagent driven extraction pathways.^59^ Region-specific adjustments balance realism: Australia ore is set to 60% (+10%) reflecting higher grades and mature mining infrastructure,^65^ while Latin American,^139^ Africa^140^ and other regions^141^ are set 5% lower for all sources to reflect development and capacity gaps. Next uncertainty concern is technology reflected by factor *β* (recovery rate). For ore, the range is around 80%–90%,^87^ while the range for brine and unconventional sources are around 55%–63%.^92^^,^^142^ Regional adjustments reflect technological capability: North American, Europe and Australia take the upper bound; Latin American, Africa, and other take the lower bound, with one exception, Latin American brine adopts the mid-point (59%) given the region’s relatively mature brine value chain.^143^ As mentioned, a normal distribution with standard deviation 20% and 10% are setting for factor *α* and *β.* This choice follows good practice guidance on uncertainty reporting: when cross-project and cross-stage variability is material, a wider distribution with Monte Carlo and sensitivity analysis provides a transparent assessment of parameter.^144^ Because resource-to-reserves conversion is subject to greater geological and project-specific unknowns, *α* is assigned the broader spread (20%), while *β* adopts a narrower one (10%). The detailed figures are shown in Table S11.

For economic (cost) uncertainty, beyond the preprocessing undertaken in the terminology stage, three coherent implementation rules are applied. First, where a deposit lacks cost information, the standard deviation for factor *α* and factor *β* are increased by 5%, reflecting that project lacking exploration. Second, for the same deposits, when AISC is inferred via the depletion curve, a tail uplift is applied to mitigate underestimation liked to grade decline, ranging from 10%–20% (Details in Section “step 4”). Third, where initial capital cost (ICAPEX) is unreported, it is approximated using category average ICAPEX to AISC rate: ore 13.8%, brine 21.35% and unconventional 21.74% (Calculation based on Tables S6–S8).

Most parameter values are drawn from the literature, with a minority based on judgment (standard deviation figures, and tail uplift range). To examine the contribution of each uncertainty to the Cumulative Availability Curve (CAC), a sensitivity analysis was conducted on all parameters. Results are presented in two groups: Physical & Technology uncertainties, which affect the Estimated Recoverable Minerals (ERM), and Economic uncertainties, which affect the All-in Cost (AIC). The former covers five variable sets: the upper and lower bounds of the triangular distribution for *Coefficient A* (ERM_Tri); the baseline value of factor *α* (ERM_ *α*); the baseline value of factor *β* (ERM_ *β*); the standard deviations assigned to *α* and *β* (ERM_Std); and the standard deviations uplift applied to deposits without cost information (MisStd). For AIC, three groups are examined: the ICAPEX-to-AISC ratios by lithium source type used when computing initial capital cost (AIC_Cpex); the tail-uplift coefficients applied when inferring AISC for deposits lacking cost data (AIC_Tail); MisStd (which influences both ERM and AIC). For each group, every variable group was perturbed six times, by ±1%, ±5, and ±10%, to quantify directional effects and elasticities, thereby revealing which assumptions most strongly influence the CAC.

### Quantification and statistical analysis

There are no quantification or statistical analyses to include in this study.

## Supplemental information

Document S1. Figures S1–S9 and Tables S1–S11, and S15

Table S12. Ranked lithium deposits based on all-in cost, with associated estimated recoverable minerals, regions, and deposit types

Table S13. Lithium ore deposit grades across countries included in this study

Table S14. Lithium brine deposit grades across countries included in this study
