# Supplementary material for: The implications of uncertainties on global lithium resources availability estimations
Source: iScience. 2026 Jan 6;29(2):114629. doi: 10.1016/j.isci.2026.114629 (PMC12859240; doi:10.1016/j.isci.2026.114629)
Supplement: Document S1. Figures S1–S9 and Tables S1–S11, and S15 [file mmc1.pdf]

iScience, Volume *29*

Supplemental information

The implications of uncertainties on global lithium resources availability estimations

Dongping Zhou, Steve Pye, Brunilde Verrier, and Paul Dodds

Supplemental Information

Section 1 – Data Collection

Table S1 Lithium conversion table.

	to Li	***to Li_2O***	***to Li_2CO_3***	***to LiOH***
from Li	1	2.153	5.325	6.048
from Li_2O	0.464	1	2.473	2.809
from Li_2CO_3	0.188	0.404	1	1.136
from LiOH	0.165	0.356	0.88	1

Table S2 USA inflation rate % and relevant adjustment factor to 2023 (2005-2024) [1].

**Year**	**Inflation Rate %**	**Adjustment factor to 2023**
**2005**	3.39	0.64
**2006**	3.23	0.66
**2007**	2.85	0.68
**2008**	3.84	0.71
**2009**	−0.36	0.70
**2010**	1.64	0.72
**2011**	3.16	0.74
**2012**	2.07	0.75
**2013**	1.46	0.76
**2014**	1.62	0.78
**2015**	0.12	0.78
**2016**	1.26	0.79
**2017**	2.13	0.80
**2018**	2.44	0.82
**2019**	1.81	0.84
**2020**	1.23	0.85
**2021**	4.70	0.89
**2022**	8.00	0.96
**2023**	4.12	1.00
**2024**	3.00	1.03

Table S3 Lithium brine resources by country. *Country codes: ISO 3166-1 alpha-3.*

Country Code	*Deposit/project name*	*Resources (Min) (Mt Li content)*	*Resources (Mean) (Mt Li content)*	*Resources (Max) (Mt Li content)*
AFG	Salt lakes-Afghanistan [2]	0.40	0.40	0.40
ARG	3Q (Tre Quebradas) [2,3]	0.75	1.03	1.30
	Candelas [4]	0.24	0.24	0.24
	Cauchari-JV project-AL [2,5]	0.90	1.04	1.18
	Cauchari-Olaroz-LA [2,6-8]	0.90	2.54	4.62
	Centenario ratones [2,9,10]	0.92	1.55	1.88
	Diablillos [2,6,11]	0.38	0.73	0.90
	Hombre Muerto North [2,12]	0.11	0.11	0.11
	Hombre Muerto West [13-15]	1.08	1.08	1.08
	Hombre Muerto-Mina Fenix [2,16]	0.84	1.08	1.33
	Kachi [2,17]	0.83	1.10	1.37
	Mariana [2,18]	0.23	0.61	0.98
	Pastos Grandes [2,19]	0.78	0.85	0.92
	Pozuelos [2,20]	0.24	0.45	0.67
	Rincon-Argusy [2,21]	0.05	0.05	0.05
	Rincon-Power Minerals [2,22]	0.01	0.02	0.03
	Rincon-Rio Tinto [2,23]	1.49	1.70	1.91
	Sal de Oro [2]	0.39	0.39	0.39
	Sal de Vida [2,6,11,24]	0.30	0.75	1.22
	Salar de Antofallo [2,25]	2.22	2.22	2.22
	Salar de Olaroz-Allkem [2,6,8,9,11,26-30]	0.20	0.83	3.50
	Salar de Pular [2,31]	0.03	0.06	0.09
	Salar de Rio Grande [2,32]	0.21	0.31	0.41
	Tolillar [33]	0.68	0.68	0.68
BOL	Remaining Unknow (USGS) [2,34]	12.80	16.95	21.10
	Uyuni [6,8,11,27-30]	5.50	8.00	10.20
CAN	Beaverhill [6]	0.59	0.59	0.59
	Fox Creek [6,8,28]	0.50	0.50	0.50
	Kindersley [35]	0.86	0.86	0.86
	Prairie [36]	0.85	0.85	0.85
	Valley View [2]	0.07	0.07	0.07
CHL	Arizaro [37]	0.47	0.47	0.47
	Atacama-ALB [38]	1.85	1.85	1.85
	Atacama-SQM [39]	8.56	8.56	8.56
	La Isla [2,40]	0.27	0.27	0.27
	Las Parinas [2,40]	0.05	0.05	0.05
	Maricunga [2,8,28,30]	0.20	0.30	0.40
	Pedernales [2,40]	0.38	0.38	0.38

	Punta Negra [2,40]	0.22	0.22	0.22
	Salar Blanco [41]	0.36	0.36	0.36
	Salar de Aguilar [2,40]	0.07	0.07	0.07
	Surire [40]	0.18	0.18	0.18
CHN	Chaerhan Lake [2]	1.35	1.35	1.35
	DXC [2]	0.90	0.90	0.90
	East Taijinair [2]	2.47	2.47	2.47
	Lakkor Tso Salt Lake [42]	0.41	0.41	0.41
	Qarhan Lake [2]	7.17	7.17	7.17
	Qinghai Yiliping [2,8,11,28-30]	0.90	1.71	2.30
	West Taijinair [2]	2.68	2.68	2.68
	Zabuye (Zhabuye) [2,6,8,11,28,29]	1.30	1.52	1.83
MNG	Chuluut area [2]	0.10	0.10	0.10
	Delgerekh area [2]	0.20	0.20	0.20
	Enkh area [2]	0.05	0.05	0.05
USA	Bonneville Salt Flats [6]	1.97	1.97	1.97
	Brawley [6]	1.00	1.00	1.00
	G. Salt Lake [2,6,8,29,30]	0.50	0.51	0.53
	Searles [2,6,30]	0.03	0.13	0.32
	Silver Peak [6,11,28,29,43]	0.05	0.21	0.30

Table S4 Lithium ore resources by country. *Country codes: ISO 3166-1 alpha-3. "0.00" < 0.004.*

Country Code	***Deposit/project name***	***Resources (Min) (Mt Li content)***	***Resources (Mean) (Mt Li content)***	***Resources (Max) (Mt Li content)***
AFG	Drumgal [2]	0.10	0.10	0.10
	Helmand Basin [6]	0.20	0.20	0.20
	Jamanak [2]	0.10	0.10	0.10
	Katawaz Basin [6]	0.01	0.01	0.01
	Parun [2]	0.03	0.03	0.03
	Pasghusta [2]	0.50	0.50	0.50
	Pasghusta lower [2]	0.06	0.06	0.06
	Paskhi [2]	0.06	0.06	0.06
	Taghawkor [6]	0.00	0.00	0.00
	Tsamgal [2]	0.06	0.06	0.06
	Yaryhgul [2]	0.10	0.10	0.10
AUS	Bald Hill [2,44]	0.03	0.08	0.12
	Buldania [2,45]	0.04	0.05	0.07
	Greenbushes [2,30,46]	0.26	1.67	3.06
	Kathleen Valley [2,47]	0.84	0.91	0.98
	Mt Cattlin [2,6,11,30,48]	0.06	0.07	0.09
	Mt Holland [2,49]	1.32	1.61	1.90
	Mt Marion [2,6,30]	0.02	0.16	0.45
	Pilgangoora [2,50]	1.53	1.68	1.83
	Wodgina [2,51]	0.92	1.17	1.41
AUT	Wolfsberg [2,6,8,27,28,30,52]	0.05	0.09	0.10
BRA	Aracuai (Cachoeira) [2,11]	0.01	0.02	0.02
	Bandeira [53]	0.25	0.25	0.25
	Grota do Cirilo [9,30,54]	0.09	0.55	0.92
	Mibra [2,28,55]	0.09	0.10	0.12
CAN	Authier [2,56]	0.10	0.13	0.15
	Barraute [6,11,30]	0.11	0.20	0.37
	Bernic [6,8,30]	0.10	0.14	0.19
	Big Bird (Curlew) [6]	0.20	0.20	0.20
	English River Greenstone [6]	0.13	0.13	0.13
	FI [6,11]	0.03	0.03	0.03
	Georgia Lake [2,57]	0.07	0.07	0.08
	Gods Lake [6]	0.03	0.03	0.03
	Irgon [2]	0.01	0.01	0.01
	James Bay [2,6,11,58]	0.13	0.29	0.62
	La Corne [6,8,27]	0.10	0.30	0.40
	La Motte [6,30]	0.02	0.52	1.02
	Lithium Two [2]	0.00	0.00	0.00

	Moblan [2,6,59]	0.04	0.14	0.28
	Moose 2 [6]	0.02	0.02	0.02
	Nama Creek [6]	0.01	0.01	0.01
	Niemi Lake [6]	0.00	0.00	0.00
	North American Lithium [60]	0.12	0.12	0.12
	PAK [2]	0.21	0.21	0.21
	Rose [2,61]	0.14	0.21	0.28
	Sep. Rapids [2,6,30]	0.07	0.07	0.07
	Seymour Lake [2]	0.03	0.03	0.03
	Sirmac Lake [6]	0.00	0.00	0.00
	Snow Lake [2,6,62]	0.03	0.04	0.05
	Tanco [11]	0.14	0.14	0.14
	Thor [6,11]	0.02	0.02	0.02
	Violet [6,11]	0.01	0.01	0.01
	Wekusko [6]	0.03	0.03	0.03
	Whabouchi [2,63]	0.26	0.30	0.33
	Yellowknife [2,6,30]	0.11	0.12	0.13
CHN	Daoxian [6,8,28]	0.20	0.20	0.20
	Heyuan [2]	0.03	0.03	0.03
	Jiajika (Giajika) [2,6,8,11,27,28,30,64]	0.20	0.47	0.78
	Jinchuan [6]	0.50	0.50	0.50
	Lijiagou [2,65]	0.07	0.12	0.18
	Maerkang [6,8,27,28,30]	0.20	0.31	0.50
	Ningdu [6]	0.50	0.50	0.50
	Xiangyuan [66]	0.12	0.12	0.12
	Yelonggou [67]	0.06	0.06	0.06
	Yichun [6,8,28,68]	0.14	0.31	0.50
COD	Kitotolo [6]	0.80	0.80	0.80
	Manono [6,8,11,27,28]	0.33	1.97	3.10
ESP	Mina Feli [6]	0.01	0.01	0.01
FIN	Keliber [2,6,11,30,69]	0.01	0.17	0.68
GHA	Ewoyaa [2,70]	0.09	0.13	0.16
IRL	Blackstairs [2]	0.00	0.00	0.00
MLI	Goulamina [71]	0.45	0.45	0.45
NAM	Karibib [2,6,11]	0.02	0.11	0.15
PRT	Alvarroes [2,72]	0.02	0.06	0.09
	Barroso [2,6,30,73]	0.01	0.06	0.13
	Montalegre [2]	0.15	0.15	0.15
RUS	Achivansky (Uchastok) [6]	0.05	0.05	0.05
	Belerechenskoe [2,6]	0.05	0.07	0.10
	Etykinskoe [6]	0.05	0.05	0.05
	Goltsovoe [2,6,28]	0.14	0.21	0.29
	Kolmorzerskoe [2,6]	0.40	0.62	0.84

	Orlovskoe [6]	0.05	0.05	0.05
	Pogranichnoe [6]	0.05	0.05	0.05
	Polmostundrovskoe [2,6,28]	0.14	0.25	0.40
	Tastyg [2,6]	0.05	0.18	0.30
	Ulug-Tanzek [2,6,28]	0.14	0.21	0.30
	Urikskoe [2,6,28]	0.14	0.21	0.30
	Vishnyakovskoe [2,6]	0.21	0.21	0.21
	Voznesenskoe [2,6]	0.14	0.31	0.48
	Zavitinskoe [2,6]	0.10	0.12	0.14
SWE	Jarkvissle [6]	0.01	0.01	0.01
	Varutrask [6]	0.01	0.01	0.01
USA	Bessemer City [6,11]	0.42	0.42	0.42
	Carolina [74]	0.15	0.15	0.15
	Cherryville [30]	0.34	0.34	0.34
	Kings Mountain Bely [6,11,30]	0.15	1.64	5.90
	N. Carolina [6,8,27,28,30]	2.60	3.80	5.50
UZB	Naukinskoe [2]	0.00	0.00	0.00
	Shavazsai [2]	0.10	0.10	0.10
ZWE	Arcadia [2,75]	0.28	0.30	0.31
	Barkam [6,11]	0.22	0.22	0.22
	Bikita [2,6]	0.06	0.11	0.17
	Kamativi [6,11]	0.28	0.28	0.28
	Masvingo [6]	0.06	0.06	0.06
	Sabi Star [76]	0.61	0.61	0.61
	Zulu [2]	0.10	0.10	0.10

Table S5 Lithium unconventional resources by countries. *Country codes: ISO 3166-1 alpha-3. "0.00" < 0.004.*

Country Code	***Deposit/project name***	***Resources (Min) (Mt Li content)***	***Resources (Mean) (Mt Li content)***	***Resources (Max) (Mt Li content)***
CAN	Clearwater [2,77]	0.41	0.49	0.56
	Exshaw [2]	0.73	0.73	0.73
	Rocky [2]	0.17	0.17	0.17
CZE	Cinovec [2,78]	0.85	1.06	1.28
DEU	Sadisdorf [2]	0.05	0.05	0.05
	Vulcan [2]	2.98	2.98	2.98
	Zinnwald [2,79]	0.14	0.29	0.43
ESP	San Jose [2,80]	0.17	0.24	0.32
FRA	Echassieres [2]	0.10	0.10	0.10
	Treguennec [2]	0.03	0.03	0.03
KAZ	Smirnovskoe, Drozhilovskoye [2]	0.04	0.04	0.04
MEX	Sonora [2,81]	0.95	1.30	1.66
	Zacatecas [2]	0.05	0.05	0.05
PER	Falchani [2,82]	0.89	0.96	1.04
SRB	Jabar [2,6,8,9,27,28,83]	0.44	0.84	1.15
USA	Clayton Valley-Cypress [2,84]	0.64	1.00	1.36
	Clayton Valley-Zues [2,85]	0.00	0.49	0.97
	Fort Cady [2,86]	0.04	0.13	0.22
	Kings Valley-Thacker Pass [2,6,9,87]	0.58	1.79	3.03
	Paradox [2,88]	0.04	0.09	0.15
	Rhyolite Ridge [2,9,89]	0.20	0.22	0.23
	Salton Sea [2,6,8,27-30]	0.51	1.12	2.00
	Smackover-Arkansas [2,6,8,27-30,90]	0.27	0.83	1.00
ZWE	Kamativi [2,6,11,91]	0.03	0.17	0.28

Table S6 Lithium brine deposits cost relevant data by countries. *Country codes: ISO 3166-1 alpha-3. "0.00" < 0.004. AISC = All-in Sustainability Cost; ICAPEX = Initial Capital Cost; LOM = Life of Mine; AP = annual production. "Sal de Vida" including Phase 1 and Phase 2 plants, LOM and AP data give separately.*

Country code	***Deposit/project name***	***AISC (2023USD /t LCE)***	***ICPEX (2023USD /t LCE)***	***LOM (Year)***	***AP (t LCE)***
AFG	Salt lakes-Afghanistan [2]	10,874			
ARG	3Q (Tre Quebradas) [3,92]	5,251	512	35	20,000
	Candelas [4]	6,483	1,311	25	14,000
	Cauchari-JV project-AL [5]	5,878	791	30	22,400
	Cauchari-Olaroz-LA [6,7,92]	5,013	415	40	40,000
	Centenario ratones [10]	5,012	417	25	14,000
	Diablillos				
	Hombre Muerto North [12]	5,191	742	30	5,000
	Hombre Muerto West [15]	8,355	522	40	26,218
	Hombre Muerto-Mina Fenix [16,92]	7,645	2,203	40	9,800
	Kachi [17]	8,707	800	25	25,000
	Mariana [18]	4,585	3,038	25	10,000
	Pastos Grandes [19]	5,540	567	41	22976
	Pozuelos				
	Rincon-Argusy [21,30]	6,868	1,583	17	10,000
	Rincon-Power Minerals [22,30]	7,565	2,191	14	7,061
	Rincon-Rio Tinto [23,30]	5,569			
	Sal de Oro [92,93]	5,776		20	25,000
	Sal de Vida [24]	6,238	603	40/ 37	15,000/ 30,000
	Salar de Antofallo				
	Salar de Olaroz-Allkem [6,26,30,92]	6,616	531	32	25,000
	Salar de Pular				
	Salar de Rio Grande				
	Tolillar [33]	6,928	778	35	28,538
BOL	Remaining Unknow (USGS)				
	Uyuni [30]	6,191			
CAN	Beaverhill				
	Fox Creek				
	Kindersley [35]	4,747	1,342	20	12,500
	Prairie [36]	3,185	2,784	20	6,000
	Valley View				
CHL	Arizaro [37]	7,131	2,048	20	25,000
	Atacama-ALB [38,92]	20,025			
	Atacama-SQM [39,92]	16,625	329	9	253,000
	La Isla				

	Las Parinas				
	Maricunga [30]	5,567			
	Pedernales				
	Punta Negra				
	Salar Blanco [41]	5,238	1,361	20	23,950
	Salar de Aguilar				
	Surire				
CHN	Chaerhan Lake [92]	6,703			
	DXC [30]	5,258			
	East Taijinair [92]	7,232			
	Lakkor Tso Salt Lake [92]	7,385			
	Qarhan Lake [92]	7,594			
	Qinghai Yiliping [92]	7,299			
	West Taijinair [92]	7,535			
	Zabuye (Zhabuye) [6,30]	5,894			
MNG	Chuluut area [2]	8,857			
	Delgerekh area [2]	8,857			
	Enkh area [2]	8,857			
USA	Bonneville Salt Flats				
	Brawley				
	G. Salt Lake				
	Searles				
	Silver Peak [6,92]	10,329			

Table S7 Lithium ore deposits cost relevant data by countries. *Country codes: ISO 3166-1 alpha-3. AISC = All-in Sustainability Cost; ICAPEX = Initial Capital Cost; LOM = Life of Mine; AP = annual production.*

Country code	***Deposit/project name***	***AISC (2023USD /t LCE)***	***ICPEX (2023USD /t LCE)***	***LOM (Year)***	***AP (t LCE)***
AFG	Drumgal [30]	9,312			
	Helmand Basin [30]	9,312			
	Jamanak [30]	9,312			
	Katawaz Basin [30]	9,312			
	Parun [30]	9,312			
	Pasghusta [30]	9,312			
	Pasghusta lower [30]	9,312			
	Paskhi [30]	9,312			
	Taghawkor [30]	9,312			
	Tsamgal [30]	9,312			
	Yaryhgul [30]	9,312			
AUS	Bald Hill [44,92]	5,506	631	3.6	22,999
	Buldania				
	Greenbushes [6,30,92]	4605			
	Kathleen Valley [47,92]	4,064	228	23	115,597
	Mt Cattlin [92]	5,493			
	Mt Holland [92]	7,878			
	Mt Marion [92]	4,834			
	Pilgangoora [92]	3,987			
	Wodgina [92]	2,914			
AUT	Wolfsberg [52]	20,973	7,661	14.6	7,744
BRA	Aracuai (Cachoeira) [92]	1,194			
	Bandeira [53]	4,117		14	24,164
	Grota do Cirilo [30,92]	4,268			
	Mibra				
CAN	Authier [56]	3,888	806	13.8	16,933
	Barraute				
	Bernic				
	Big Bird (Curlew)				
	English River Greenstone				
	FI				
	Georgia Lake [57]	1,487	1,497	9	14,838
	Gods Lake				
	Irgon				
	James Bay [6,58]	3,026	437	19	45,998
	La Corne [6]	5,302			
	La Motte				

	Lithium Two				
	Moblan [59]	3,977	750	21	44,514
	Moose 2				
	Nama Creek				
	Niemi Lake				
	North American Lithium [60,92]	4,525		20	28,198
	PAK				
	Rose [61]	4,621	916	17	30,235
	Sep. Rapids				
	Seymour Lake				
	Sirmac Lake				
	Snow Lake				
	Tanco [30]	5,038			
	Thor				
	Violet				
	Wekusko				
	Whabouchi [63]	6,120	458	24	32,851
	Yellowknife [30]	8,782			
CHN	Daoxian				
	Heyuan				
	Jiajika (Giajika) [30,92]	4,136			
	Jinchuan				
	Lijiagou [92]	2,677			
	Maerkang [30]	5,919			
	Ningdu				
	Xiangyuan [92]	4,798			
	Yelonggou [92]	2,457			
	Yichun [92]	1,211			
COD	Kitotolo				
	Manono [30]	9,208			
ESP	Mina Feli				
FIN	Keliber [69]	11,661	3,158	6	32,309
GHA	Ewoyaa [70]	3,654	365	12	42,475
IRL	Blackstairs [2]	7,390			
MLI	Goulamina [71,92]	3,032	1,615	21	10,772
NAM	Karibib [30]	8,583			
PRT	Alvarroes [92]	4,505			
	Barroso [30,73]	5,437	770	14	25,979
	Montalegre [2]	7,345			
RUS	Achivansky (Uchastok) [30]	13,466			
	Belerechenskoe [30]	13,466			
	Etykinskoe [30]	8,162			
	Goltsovoe [30]	13,466			

	Kolmorzerskoe [30]	13,466			
	Orlovskoe [30]	13,466			
	Pogranichnoe [30]	13,466			
	Polmostundrovskoe [30]	13,466			
	Tastyg [30]	13,466			
	Ulug-Tanzek [30]	13,466			
	Urikskoe [30]	13,466			
	Vishnyakovskoe [30]	13,466			
	Voznesenskoe [30]	13,466			
	Zavitinskoe [30]	13,466			
SWE	Jarkvissle				
	Varutrask				
USA	Bessemer City				
	Carolina [74]	8,271	3,826	11	26,400
	Cherryville [30]	5,038			
	Kings Mountain Bely [30]	7,020			
	N. Carolina [30]	12,792			
UZB	Naukinskoe [30]	9312			
	Shavazsai [30]	9312			
ZWE	Arcadia [75,92]	2,959	300	18.3	39,321
	Barkam				
	Bikita [30,92]	3,865			
	Kamativi				
	Masvingo				
	Sabi Star [92]	2,811			
	Zulu				

Table S8 Lithium unconventional deposits cost relevant data by countries. *Country codes: ISO 3166-1 alpha-3. AISC = All-in Sustainability Cost; ICAPEX = Initial Capital Cost; LOM = Life of Mine; AP = annual production.*

Country code	***Deposit/project name***	***AISC (2023USD /t LCE)***	***ICPEX (2023USD /t LCE)***	***LOM (Year)***	***AP (t LCE)***
CAN	Clearwater [77]	8,654	1,887	50	29,375
	Exshaw				
	Rocky				
CZE	Cinovec [78]	5,367	1,037	25	25,860
DEU	Sadisdorf				
	Vulcan [2,94]	7,119	1,920	30	27,273
	Zinnwald [2,79]	10,682	1,253	45	31,163
ESP	San Jose [80]	9,965	1,343	26	17,142
FRA	Echassieres				
	Treguennec				
KAZ	Smirnovskoe, Drozhilovskoye				
MEX	Sonora [81]	5,454	1,615	20	30,045
	Zacatecas				
PER	Falchani [82]	5,770	823	43	70,419
SRB	Jabar				
USA	Clayton Valley-Cypress [84]	12,592			
	Clayton Valley-Zues [85]	11,904			
	Fort Cady				
	Kings Valley-Thacker Pass [87]	8,579	1,557	40	66,800
	Paradox				
	Rhyolite Ridge [89]	4,101	1,640	26	21,951
	Salton Sea				
	Smackover-Arkansas				
	Kamativi				

__Table S9 Regional classification and countries included in this study.__ Country codes: ISO 3166-1 alpha-3.

Regional abbreviation	***Regional full name***	***Countries included in this study (Code)***
AFR	Africa	COD, GHA, MLI, NAM, ZWE
AUS	Australia	AUS
CHN	China	CHN
EU	Europe	AUT, CZE, DEU, ESP, FIN, IRL, PRT, SRB, SWE
LATAM	Latin America	ARG, BOL, BRA, CHL
NAM	North America	CAN, USA
OTH	Other countries	AFG, KAZ, MNG, RUS, UZB

__Table S10 Twelve distinct analytical groups for "Step 1".__ Regional abbreviations follow Table S9. "Uncon" denotes unconventional lithium sources treated collectively across regions.

**Resource types**	**Existing Regions**
Ore	AFR, AUS, CHN, EU, LATAM, NAM, OTH
Brine	CHN, EU, LATAM, NAM
Uncon	All

Table S11 Factors to calculate Estimated Recoverable Minerals (ERM) by regions. *Regional abbreviations follow Table S9*

Region	***Lower Coefficient A***	***Median Coefficient A***	***Higher Coefficient A***	***Factor α for Ore***	***Factor α for Brine & Uncon***	***STD for Factor α***	***Factor β for Ore***	***Factor β for Brine & Uncon***	***STD for Factor β***
LATAM	78%	100%	125%	45%	40%	20%	80%	59%	10%
NAM	68%	100%	146%	50%	45%	20%	90%	63%	10%
AUS	68%	100%	134%	60%	45%	20%	90%	63%	10%
CHN	33%	100%	108%	50%	45%	20%	85%	59%	10%
EU	82%	100%	122%	50%	45%	20%	90%	63%	10%
AFR	63%	100%	126%	45%	40%	20%	80%	55%	10%
OTH	22%	100%	121%	45%	40%	20%	80%	55%	10%

Section 2 – Flow chart for methodology

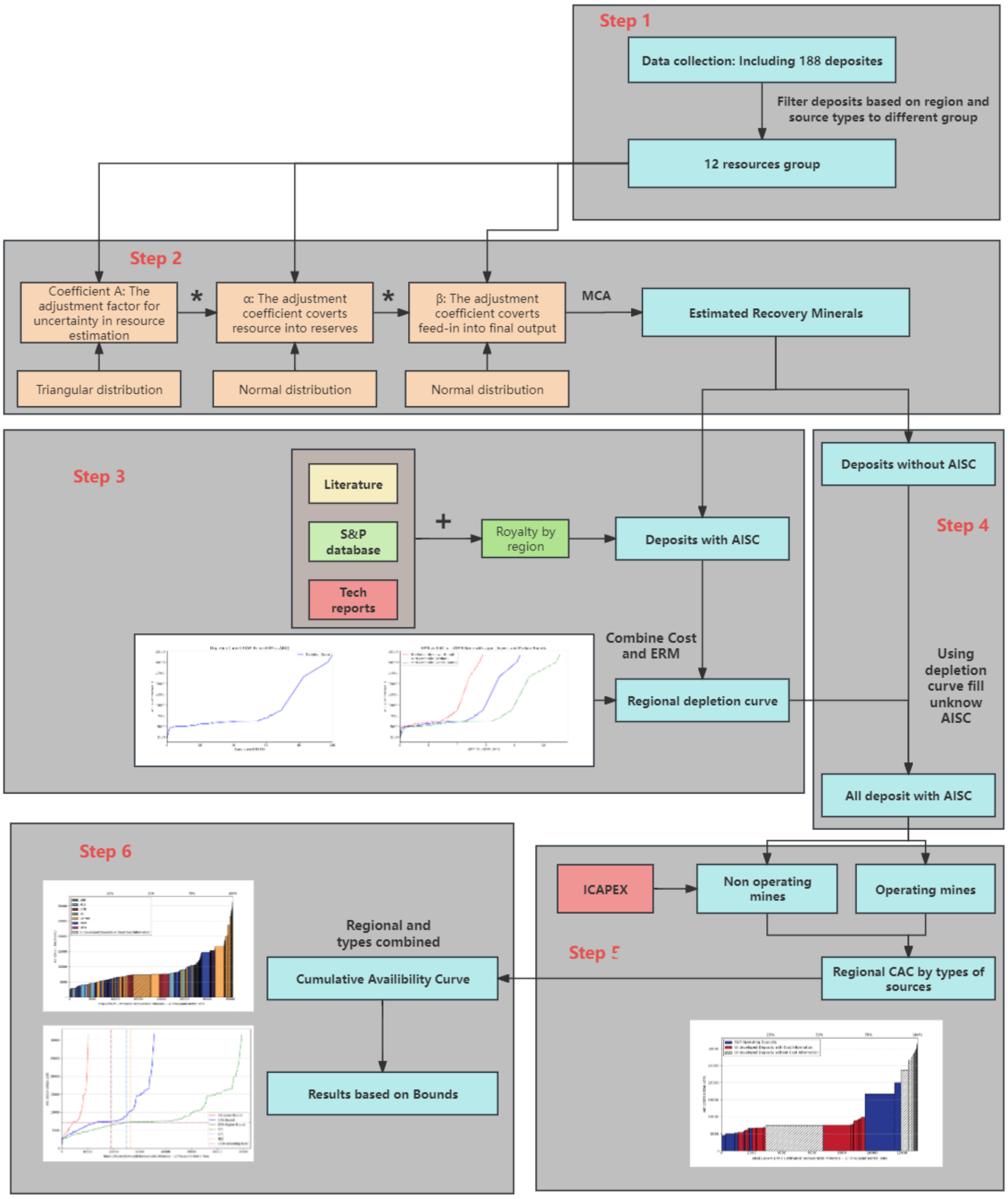

Figure S1 Flow chart for the "Six-step method for building a Cumulative Availability Curve (CAC)".

Section 3 – Detailed figures and tables of the results

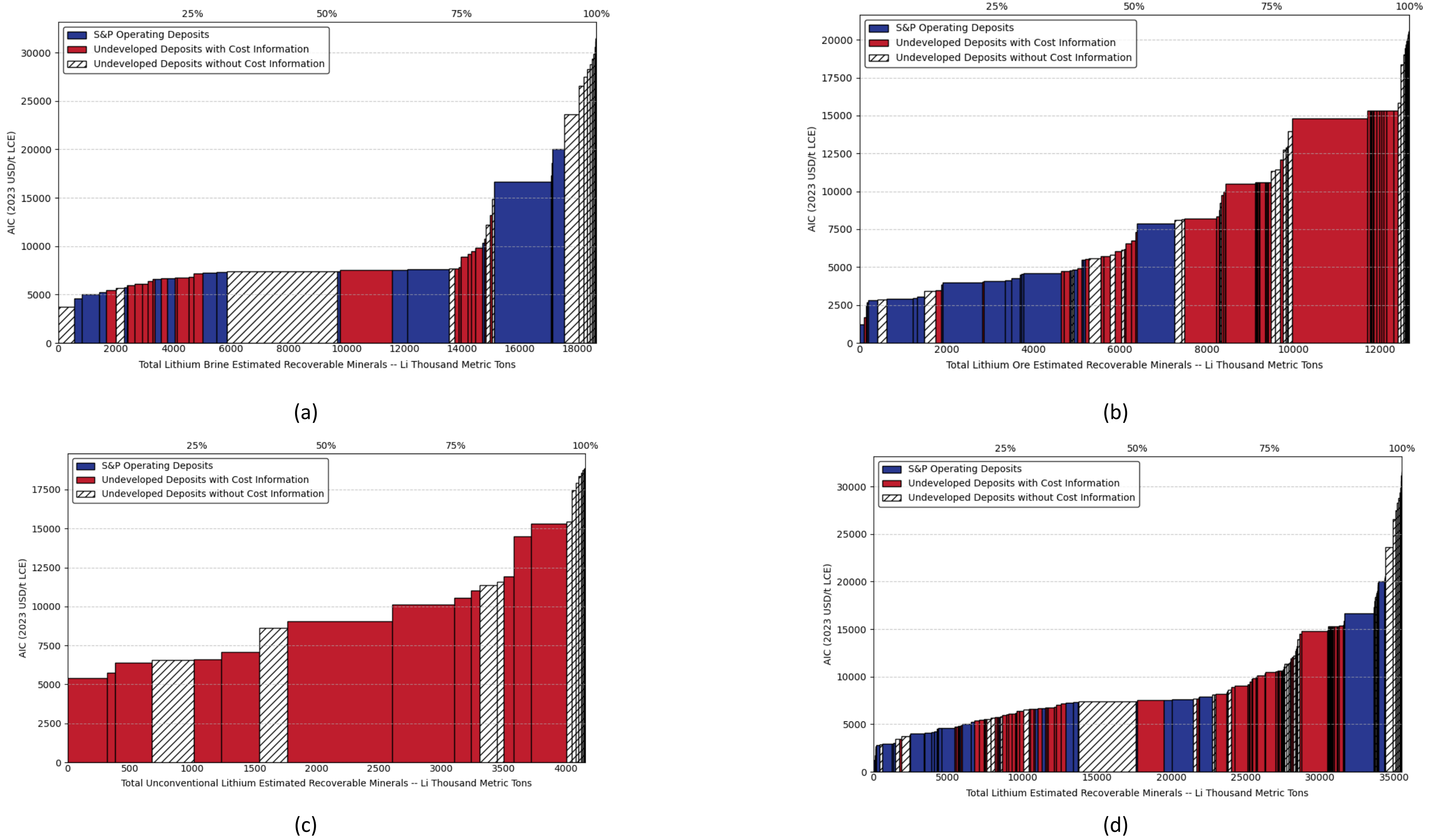

Figure S2 Global lithium CAC separated by operating status. *(a) for brine (b) for ore (c) for unconventional source (d) for total lithium sources. Related to Figure 3-6.*

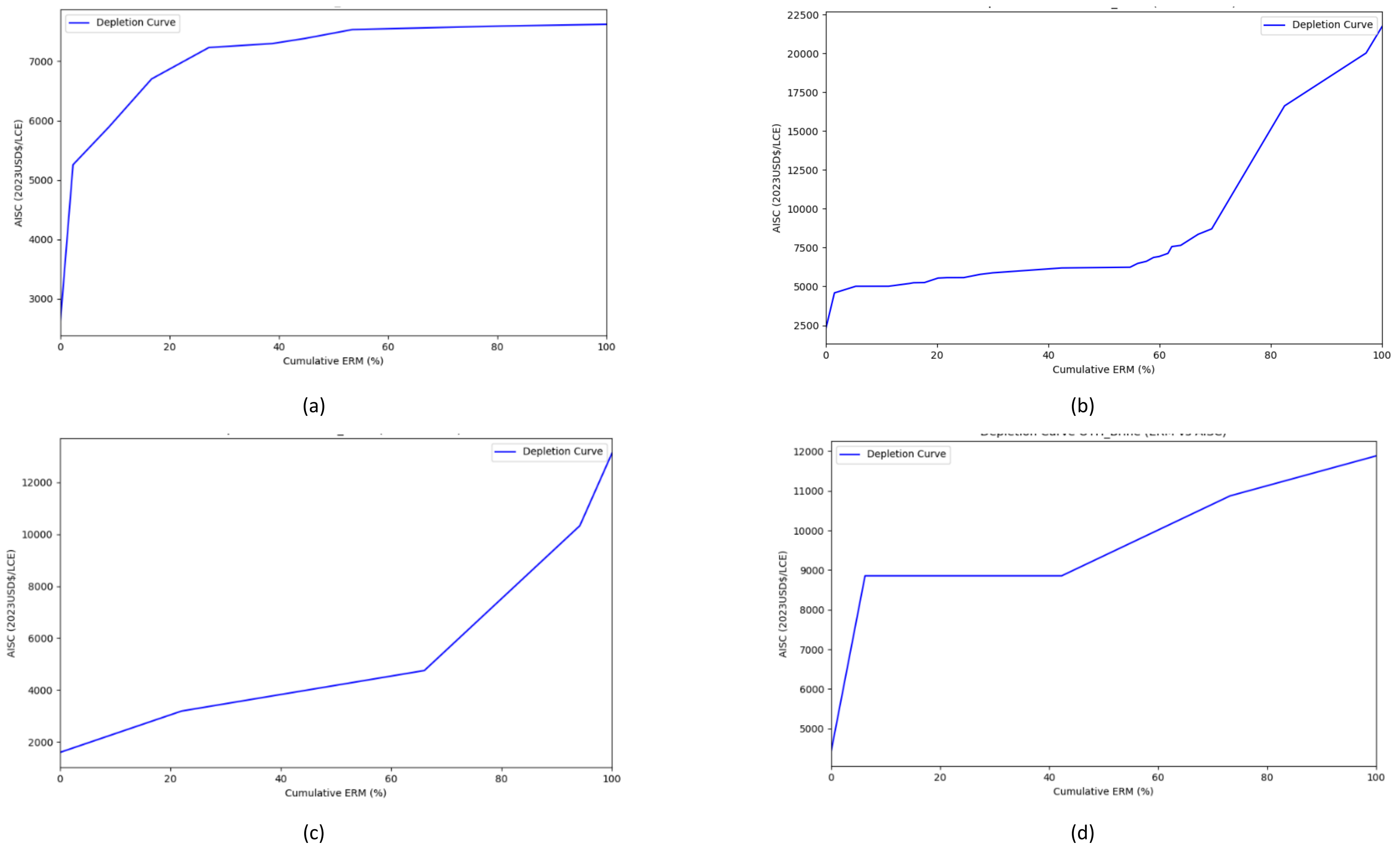

Figure S3 Lithium depletion curve for brine with cost information. *(a) for China (b) for Latin America (c) for North America (d) for other regions. Related to Figure 3.*

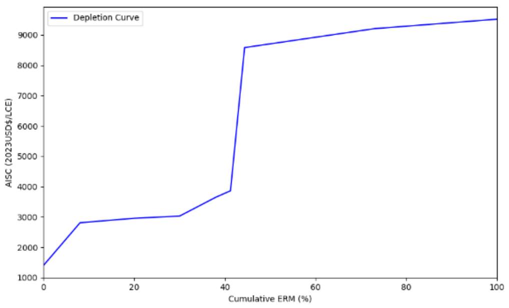

(a)

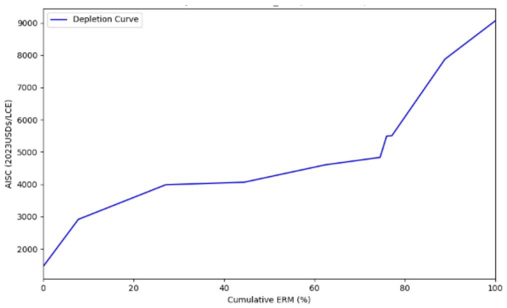

(b)

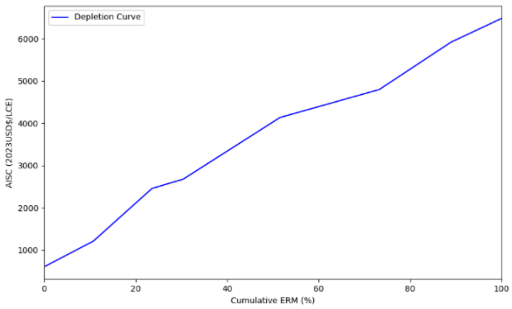

(c)

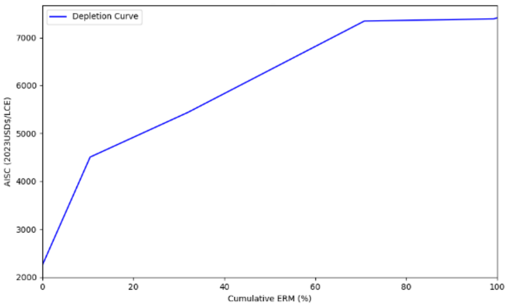

(d)

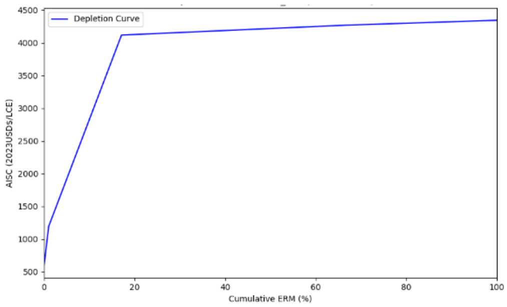

(e)

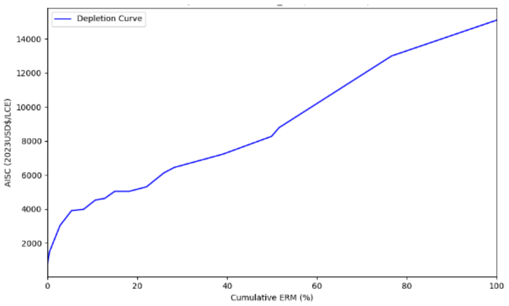

(f)

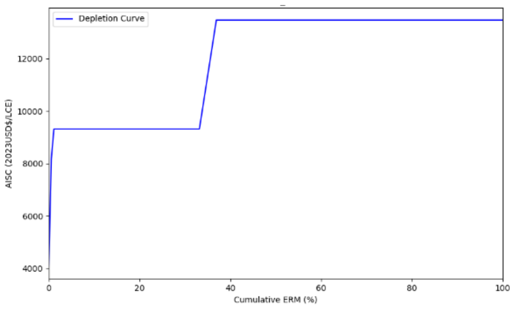

(g)

Figure S4 Lithium depletion curve for ore with cost information. *(a) for Africa (b) for Australia (c) for China (d) for Europe (e) for Latin America (f) for North America (g) for other regions. Related to Figure 4.*

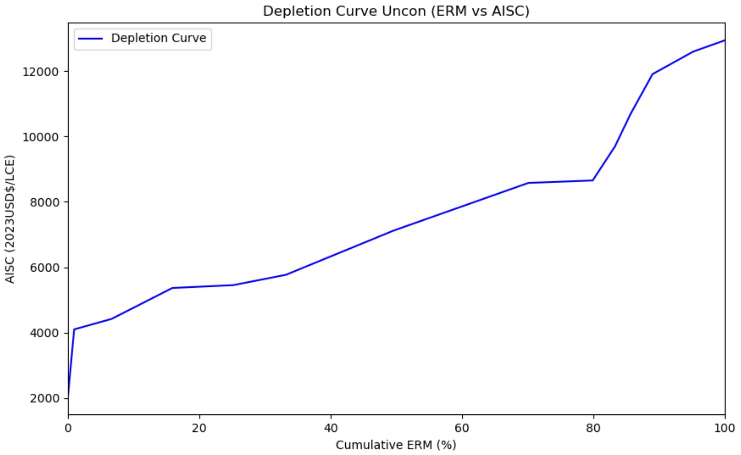

Figure S5 Lithium depletion curve for unconventional source. *Related to Figure 5.*

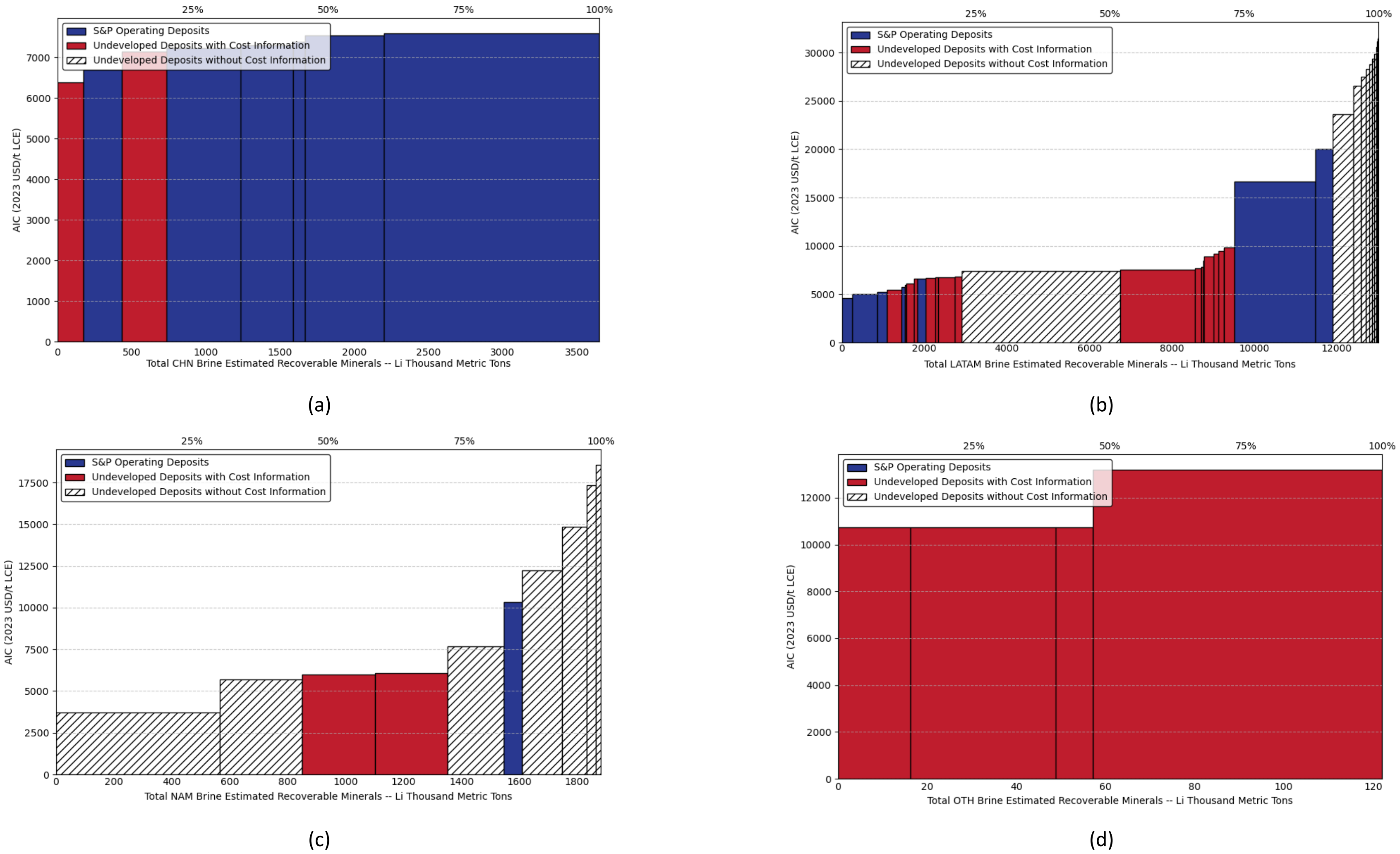

Figure S6 Regional lithium CAC from brine. *(a) for China (b) for Latin America (c) for North America (d) for other regions. Related to Figure 3.*

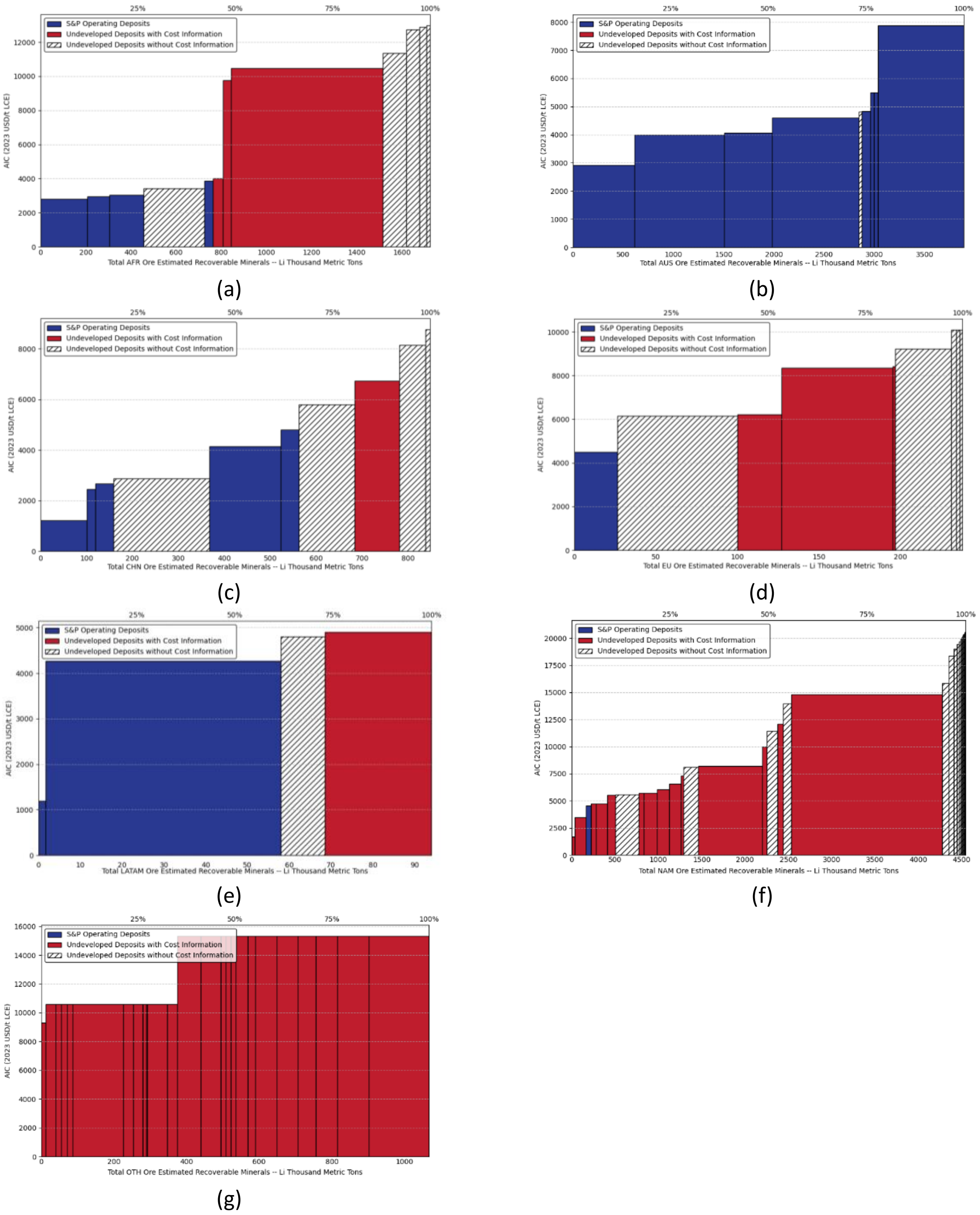

Figure S7 Regional lithium CAC from ore. *(a) for Africa (b) for Australia (c) for China (d) for Europe (e) for Latin America (f) for North America (g) for other regions. Related to Figure 4.*

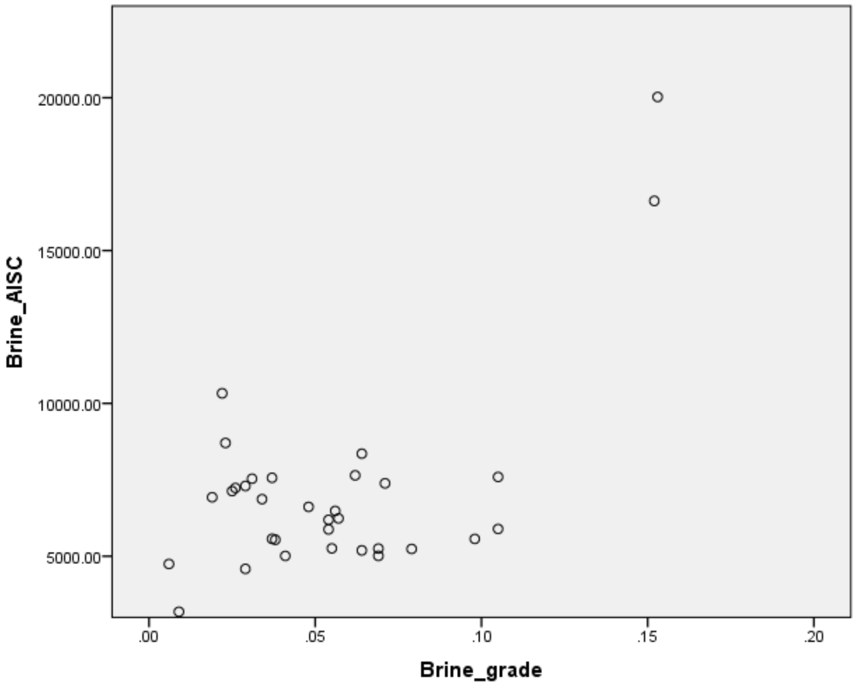

(a)

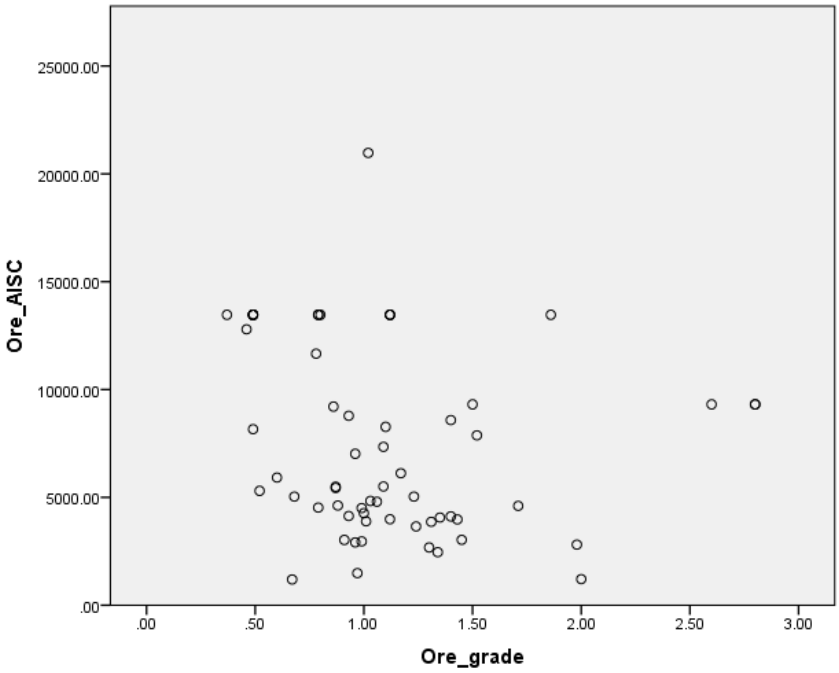

(b)

Figure S8 Relationship between lithium deposit grade and AISC. *(a) for ore (b) for brine. No clear relationship between variables.*

Section 4 – A brief introduction to Morphological Analysis and scenarios samples

Morphological analysis is a structured exploratory technique that systematically maps the key dimensions and plausible states of a complex system, enabling the identification of internally consistent combinations of future conditions[95-97]. In the context of the lithium value chain, it provides a transparent way to organise and assess uncertainties that stem from external drivers beyond addressed in the Cumulative Availability Curve (CAC) construction. By decomposing the value chain into critical parameters, morphological analysis helps to illustrate how diverse sources of uncertainty may interact and propagate. By applying morphological analysis to develop two simple scenarios, Green Leap (GL) and Fragmented Transition (FT), this approach demonstrates how CAC derived data can support scenario building and interact with uncertainties arising from external factors shaping the lithium value chain.

With in the morphological analysis framework[96], the first step following the definition of the definition of the scenario field is the identification of key factors. Through a review of the literature, six parameters relevant to the lithium value chain uncertainty were identified: non-energy lithium consumption[98,99], New Energy Vehicles (NEVs) demand[100,101], battery technology development[102,103], lithium supply chain robustness[104,105], secondary supply and storage use[98,106], climate policy ambition[107,108]. These factors collectively influence the balance of between lithium supply and demand across extraction, utilisation and recycling stages.

The subsequent step involves the analysis of key factors, which entails the systematic definition of “hypotheses” within the morphological box. Each factor is described through two or three plausible developmental settings, capturing distinct trajectories that may shape the evaluation of the lithium value chain. The full settings is presented in S3-Table 1. Finally, the combination of compatible “hypothesis” bundles enables the construction of internally consistent scenarios. On this basis, two illustrative scenarios are developed to support the interpretation and application of the morphological analysis: Green Leap (GL) and Fragmented transition (FT). The storyline of these two scenarios can be summarised as:

Green Leap:

The Green Leap scenario reflects a world in which countries work closely together to advance economic development and climate ambitions. Strong cooperation fuels global growth, which in turn drives a pronounced rise in the demand of lithium. Open trade and improving industrial capacity allow lithium to be integrated more widely across value chain. Rapid progress in lithium-ion battery technologies, accelerates the electrification of transport and expands the use of stationary storage systems. Recycling techniques also mature steadily and become economically viable in several applications, providing an increasingly valuable secondary source. Although lithium supply expands, the strengthening of ESG requirements slows the rate at which new projects can proceed. Overall, Green Leap portrays a coordinated, technology-driven pathway in which climate cooperation and innovation reinforce a rapid transition.

Fragmented Transition

The Fragmented Transition scenario describes a world where geopolitical tensions intensify, and trade restrictions become more common. Countries increasingly view lithium as a strategic mineral, and the resource nationalism uprising. Despite this conflict context, governments continue to pursue emissions reduction based on the published National Determined Contribution (NDC). Global economic continues to exhibit a trend of steady growth, impacting both non-energy lithium and road transport demand. The spread of lithium battery technologies is uneven: only a few countries with both resources and domestic markets establish integrated supply chains, while others turn to alternative low-carbon technologies. Recycling expands rapidly, and second-life use of EV batteries becomes more widespread as a response to constrained primary supply.

Overall, the two scenarios point to markedly different development pathways. Under the green Leap scenario, global cooperation steers the system towards a more efficient trajectory. Lithium resources are developed widely with ESG constraints, guided primarily by cost considerations, and can circulate freely

across regions. Mining and refining activities may remain relatively concentrated, supported by open trade and coordinated industrial development, in contrast, the Fragmented Transition scenario reflects a world in which countries prioritise the development of their own lithium deposits and invest in domestic refining capacity. Regions with limited lithium availability are likely to channel greater effort into alternative low-carbon options, such as hydrogen technologies, which rely less on lithium. Recycling also becomes a strategic focus, with governments treating secondary supply as an essential component of supply-chain security. The contrast between these two scenarios highlights how restriction on lithium trade may reshape the global landscape of lithium supply and alter the pathways through which the value chain develops.

Building on the qualitative scenarios developed through the morphological framework, these storylines can be translated into numerical assumptions that are readable by Integrated Assessment Models (IAMs). Parameter values are derived from the literature and adjusted to reflect the characteristics of scenarios. For example, Knehr et al. (2003)[109] report lithium intensities for lithium-ion batteries are range from 91-121 g/kWh. On this basis, different learning trajectories can be assigned across scenarios for lithium material intensity per vehicle, thereby capturing uncertainty in future technology development. Among all factors, the most pronounced data gaps related to the availability of lithium on the supply side, which is the focus of this study. The Cumulative Availability Curve (CAC) is used to derive regional production costs and extractable quantities. By specifying a base-year production level and an upper bound on production growth rate in subsequent years, a consistent set of supply-side inputs is produced for use in modelling simulations. Scenario descriptions subsequently inform further constraints on these pathways, while other uncertainties influence effective availability through the optimisation process. A schematic illustration of the CAC and scenario linkage, using ESG restriction and geopolitical risk as examples, is presented in Table S15.

Table S15 Morphological table of scenario development.

**Non-energy lithium consumption**	**Moderate Growth: Under Geopolitical Constraints**<br>With moderate economic expansion and persistent geopolitical risks, demand for non-energy lithium applications grows slowly and remains somewhat constrained. Limited cross-border cooperation reduces investment certainty and slows the uptake of lithium in industrial, chemical and consumer-product uses, leading to a relatively subdued trajectory for non-energy consumption.<br>FT	**Rapid Expansion: Under Global Economic Cooperation**<br>Strong international cooperation supports robust economic growth, stimulating rapid increases in non-energy lithium consumption. Broad improvements in industrial activity, material innovation and global trade facilitate accelerated uptake of lithium across chemical, industrial and consumer sectors. As a result, non-energy demand becomes an increasingly important driver of total lithium use.<br>GL	
**New energy vehicles (NEVs) demand**	**Low Growth: Weak Transport Demand and Limited NEV uptake**<br>Global economic performance weakens in the face of sustained shocks, leading to a subdued level of road transport demand. Environmental concerns become less salient as households and governments prioritise economic stability, resulting in slow uptake of NEVs. Penetration rates remain particularly low in developing economies where affordability and infrastructure constraints persist.	**Moderate Growth: Steady Expansion and Rising NEV Penetration**<br>The global economy continues to grow, but the pace of expansion slows under persistent trade frictions. Road transport demand follows a moderate trajectory, and environmental awareness gains broad societal acceptance. NEV uptake makes tangible progress; however, this advancement is largely confined to a small number of countries with strong domestic industrial capabilities. Heightened resource nationalism and restricted trade flows limit the diffusion of NEV technologies and prevent the formation of broader comparative advantages, resulting in uneven adoption across regions.<br>FT	**High Growth: Rapid Transport Demand Coupled with Accelerated NEV Uptake**<br>International cooperation strengthens supporting robust economic growth and rising living standards. Transport demand accelerates rapidly. Rapid advances in clean technologies and heightened environmental awareness drive a sharp increase in NEV penetration, alongside widespread adoption of electrified public transport.<br>GL

**Battery technology development**	**Limited Progress: Slow Innovation under Constrained R&D**<br>Economic stagnation constrains investment in research and development, leading to slow innovation and limited technological advancement. Reductions in battery costs progress only marginally, while improvements in pack-level energy density and material intensity (measured per kWh) advance at a similarly sluggish pace.	**Moderate Advancements: Diversified Technological Pathways**<br>Governments uphold their domestic emission reduction targets, providing steady momentum for transport sector transitions. However, restrictions on lithium trade and broader geopolitical risks heighten concerns over supply chain security, promoting countries t pursue more diversified technological pathways. Therefore, multiple automotive powertrain options develop in parallel. progress in lithium-ion battery technologies remains stable, but not transformative.<br>FT	**Breakthrough Innovation: Rapid Advances Driven by Global Cooperation**<br>Under conditions of strong international cooperation and a shared commitment to ambitious decarbonisation goals, lithium -ion batteries emerge as the central technological solution and experience rapid, transformative progress. Significant improvements are achieved in production costs, pollutant emissions, pack size and metal intensity, accelerating their competitiveness across transport segments. Complementary battery technologies for buses, trucks and other heavy-duty applications also advance swiftly, reinforcing lithium-ion batteries as a primary driver of the transport sector's energy transition.<br>GL
**Lithium supply chain robustness**	**Constrained Supply: Geopolitical Restrictions and Domestic-First Expansion**<br>Heightened geopolitical tensions and resource nationalism lead countries to prioritise domestic development of mining and processing capacity. Resource-rich nations gain a strategic advantage, while cross-border flows of lithium raw materials and intermediates diminish due to export controls, investment restrictions and fragmented trade relations. This inward-looking expansion results in a more regionally segmented supply landscape, amplifying vulnerabilities to localised disruptions and reducing the overall resilience of the global lithium supply chain.<br>FT	**Stable Supply with High Demand Pressure: ESG Constraints on Expansion**<br>Lithium supply grows at a steady pace, supported by stable demand and ongoing expansion of existing mining and refining capacity. However, the development of new projects progresses only slowly under increasingly stringent ESG requirements, contributing to higher overall extraction costs and modestly constraining project approval, particularly in regions with weaker ESG performance.<br>GL	**Disordered Expansion: Rapid Demand Growth and Lagging Regulatory Frameworks**<br>Lithium demand rises sharply, driven by both energy and non-energy sectors requirements. However, regulatory frameworks and ESG oversight evolve slowly, and international coordination remains limited. As a results, lithium extraction enters a phase of disordered expansion, characterised by uneven project development and inconsistent operational standards. The absence of a stable and well-regulated market contributes to pronounced price volatility, preventing supply and demand from reaching a sustainable equilibrium.

**Secondary supply and storage use**	**Moderate Recycling and High Storage Uptake**<br>Under the combined pressure of climate targets and rising environmental awareness, lithium recycling technologies achieve steady progress and begin to operate at cost-competitive levels in selected applications. Demand for stationary storage continues to grow in line with broader electrification trends, while the share of end-of-life EV batteries repurposed for storage increases at a stable pace.<br>GL	**Recycling Expansion Under Trade Constraints**<br>In response to lithium trade restrictions and the need to strengthen supply-chain security, countries accelerate the development of domestic recycling capacity, reducing costs to commercially viable levels and enabling secondary supply to act as an important buffer during import disruptions. However, despite rapid growth in recycling, secondary supply remains insufficient to offset limitations in primary production. As a result, the deployment of stationary storage expands only slowly, even though a substantial share of retired EV batteries is channelled into second-life applications.<br>FT	
**Climate policy ambition**	**National Target Compliance**<br>Countries pursue their own Nationally Determined Contributions (NDCs) and implement domestic mitigation policies within their respective institutional frameworks. While emissions reduction efforts continue, the absence of robust international coordination limits policy alignment.<br>FT	**Cooperative Action Towards the 2 °C Target**<br>Under strong international collaboration, countries align their policy frameworks and collectively advance the mitigation efforts required to meet the Paris Agreement's 2 °C goal.<br>GL	

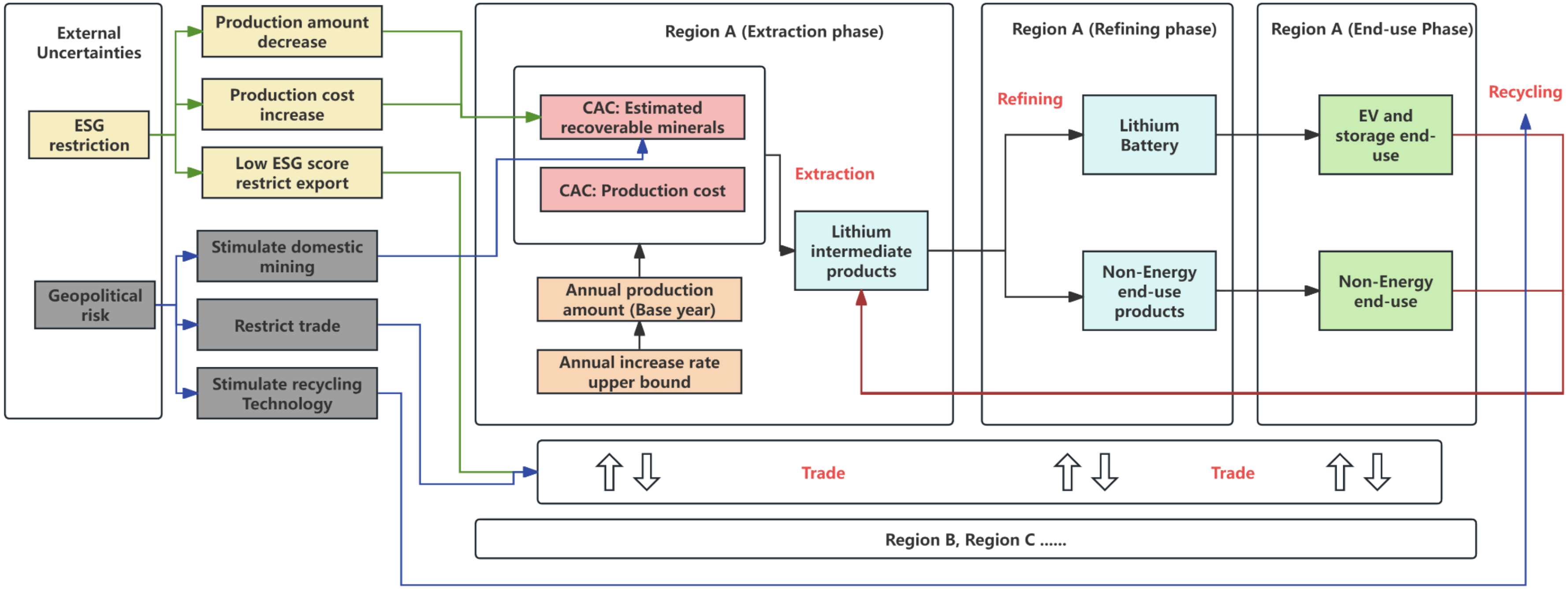

Figure S9 Linkage Between External Uncertainties and Scenario Formation. *Using ESG restriction (Green lines) and Geopolitical risk (Blue lines) as examples.*

Supplemental references

S1. The World Bank. (n.d.). Inflation, consumer prices (annual %) - United States.

S2. Fleming, M., Kannan, S.G., and Eggert, R. (2024). Long-run availability of mineral resources: The dynamic case of lithium. Resources Policy *97*, 105226.

S3. NeoLithium (2021). Tres Quebradas (3Q) Lithium Project. https://minedocs.com/21/3Q_(Tres_Quebradas)-CP-012021.pdf.

S4. Galan (2021). Excellent Preliminary Economic Assessment Results for Candelas Project in Catamarca, Argentina. https://wcsecure.weblink.com.au/pdf/GLN/02459769.pdf.

S5. AdvantageLithium (2019). Advantage Lithium Corp. Announces Positive Pre-Feasibility Study Results For The Cauchari JV. https://www.newswire.ca/news-releases/advantage-lithium-corp-announces-positive-pre-feasibility-study-results-for-the-cauchari-jv-826992858.html.

S6. Ambrose, H., and Kendall, A. (2020). Understanding the future of lithium: Part 1, resource model. Journal of Industrial Ecology *24*, 80-89. https://doi.org/10.1111/jiec.12949.

S7. LithiumAmericas (2020). NI 43 – 101 TECHNICAL REPORT Updated Feasibility Study and Mineral Reserve Estimation to Support 40,000 tpa Lithium Carbonate Production at the Cauchari-Olaroz Salars, Jujuy Province, Argentina https://s203.q4cdn.com/736266488/files/doc_downloads/our_projects/cauchari_olaroz/2020-10-19-Technical-Report.pdf.

S8. Mohr, S.H., Mudd, G.M., and Giurco, D. (2012). Lithium Resources and Production: Critical Assessment and Global Projections. Minerals *2*, 65-84.

S9. Bowell, R.J., Lagos, L., de los Hoyos, C.R., and Declercq, J. (2020). Classification and characteristics of natural lithium resources. Elements *16*, 259-264.

S10. Eramet (2023). Eramet in Argentina Centenario-Ratones Lithium Project. https://www.eramet.com/wp-content/uploads/2023/04/2023-03-08-Eramet_Centenario_Investors-Presentation.pdf.

S11. Kesler, S.E., Gruber, P.W., Medina, P.A., Keoleian, G.A., Everson, M.P., and Wallington, T.J. (2012). Global lithium resources: Relative importance of pegmatite, brine and other deposits. Ore Geology Reviews *48*, 55-69. https://doi.org/10.1016/j.oregeorev.2012.05.006.

S12. KnightPiesold (2019). NI 43-101 PRELIMINARY ECONOMIC ASSESSMENT REPORT for the HOMBRE MUERTO NORTE PROJECT SALTA PROVINCE, ARGENTINA https://www.lithiumsouth.com/wp-content/uploads/HMN-Final-Report-190808.pdf.

S13. Galan (2023). Phase 2 DFS Confirms Tier One Status of Hombre Muerto West (HMW) Lithium Brine Project in Argentina. https://wcsecure.weblink.com.au/pdf/GLN/02720109.pdf.

S14. Galan (2023). Phase 1 of Hombre Muerto West (HMW) DFS Delivers Compelling Economic Results for Accelerated Production. https://wcsecure.weblink.com.au/pdf/GLN/02682245.pdf.

S15. Galan (2024). Hombre Muerto Project. https://galanlithium.com.au/projects/hombre-muerto-west/.

S16. Integral (2023). Resource and Reserve Report Pre-Feasibility Study Salar del Hombre Muerto. https://arcadiumlithium.com/wp-content/uploads/2024/07/2023-Livent-Resource-and-Reserve-Report-Salar-del-Hombre-Muerto.pdf.

S17. LakeResources (2023). Lake Resources Kachi Project Phase One Definitive Feasibility Study.

https://lakeresources.com.au/wp-content/uploads/2023/12/lke_kachi-dfs_19-dec-23.pdf.
S18. Advisian (2018). Preliminary Economic Assessment of the Marana Lithium Brine Project. https://minedocs.com/21/Mariana-PEA-11152018.pdf.
S19. LithiumAmericas (2023). Lithium Resources Update Pastos Grandes Project Slata Provice, Argentina. https://s203.q4cdn.com/736266488/files/doc_downloads/2023-04-30-Technical-Report.pdf.
S20. HainsEngineering (2018). Technical Report on the Salar De Pozuelos Project, Salta Province, Argentina Prepared for Lsc Lithium Corporation. https://docslib.org/doc/12889719/technical-report-on-the-salar-de-pozuelos-project-salta-province-argentina-prepared-for-lsc-lithium-corporation.
S21. Argosy Minerals Limited (2018). Argosy Delivers Exceptional PEA Results For Rincon Lithium Project. https://announcements.asx.com.au/asxpdf/20181128/pdf/440q663bmjz5wk.pdf.
S22. PowerMinerals (2023). PEA delivers outstanding project economics for battery-grade lithium carbonate operation at Rincon salar. https://www.powerminerals.com.au/site/pdf/192380b1-cbf3-43a7-b42b-95f89b6fd414/Update-Rincon-PEA-delivers-outstanding-project-economics-for-batterygrade-lithium-carbonate-operation-at-Rincon-salar.pdf.
S23. Rio Tinto (2024). Rincon Project Mineral Resources and Ore Reserves https://editorial.northernminergroup.com/wp-content/uploads/2024/12/rio-tinto-rincon.pdf.
S24. Montgomery & Associates Consultores Limitada (2023). SEC Technical Report Summary Sal de Vida Lithium Brine Project. https://minedocs.com/27/Sal-de-Vida-TRS-11152023_compressed.pdf.
S25. Select Lithium (2016). Salar de Antofalla Project. https://selectlithium.com/salar-de-antofalla/.
S26. Allkem (2023). Olaroz Lithium Facility NI 43-101 Technical Report - Feasibility Study. https://www.scribd.com/document/792725155/1698636645-olaroz-lithium-facility-ni-43-101-technical-report-feasibility-study-final.
S27. Evans, R.K. (2008). An abundance of lithium. Santiago: World Lithium *1*, 1-17.
S28. Gruber, P.W., Medina, P.A., Keoleian, G.A., Kesler, S.E., Everson, M.P., and Wallington, T.J. (2011). Global Lithium Availability. Journal of Industrial Ecology *15*, 760-775. https://doi.org/10.1111/j.1530-9290.2011.00359.x.
S29. Tahil, W. (2008). The trouble with lithium 2: Under the microscope. Meridian international research, 1-54.
S30. Yaksic, A., and Tilton, J.E. (2009). Using the cumulative availability curve to assess the threat of mineral depletion: The case of lithium. Resources Policy *34*, 185-194. https://doi.org/10.1016/j.resourpol.2009.05.002.
S31. Minerals , , P. (n.d.). Pular Lithium Project. https://www.powerminerals.com.au/site/projects/lithium-brine-project/pular-lithium-project.
S32. Pursuit Minerals (2024). Rio Grande Sur Lithium Project. https://pursuitminerals.com.au/rio_grande_sur/.
S33. Ausenco (2023). Tolillar Project NI 43-101 Technical Report Update on Preliminary Economic Assessment https://minedocs.com/25/Tolillar-PEA-8102023.pdf.

S34. USGS (2024). Mineral Commodity Summaries 2024. https://pubs.usgs.gov/periodicals/mcs2024/mcs2024.pdf.

S35. Sproule (2022). NI 43-101 Technical Report: Resource Assessment of the Kindersley Lithium Project in Saskatchewan, Canada for Grounded Lithium Corp. https://groundedlithium.com/wp-content/uploads/2022/12/115092.GroundedLithium.FinalReportRev1.pdf.

S36. Arizona Lithium (2023). Prairie Lithium PFS Confirms Extremely Low Operating Costs of $2,819 USD Per Tonne. https://www.arizonalithium.com/wp-content/uploads/2024/01/02758730.pdf.

S37. Lithium Chile (2024). Lithium Chile Announces Pre-Tax NPV of US$3,853,000,000 and Pre-Tax IRR of 42.1% from Pre-Feasibility Study on Arizaro Project. https://lithiumchile.ca/wp-content/uploads/2024/07/July-23-2024-LITHIUM-CHILE-ANNOUNCES-PRE-TAX-NPV-OF-US3853000000-AND-PRE-TAX-IRR-OF-42.1-FROM-PRE-FEASIBILITY-STUDY.pdf.

S38. Albemarle (n.d.). Lithium Extraction at Salar de Atacama. https://www.albemarle.com/cl/en/what-we-offer/reliable-supply/salar-de-atacama.

S39. WSP (2022). Technical Report Summary: Operation Report Salar De Atacama. https://minedocs.com/22/Salar-de-Atacama-TR-042022.pdf.

S40. Cabello, J. (2021). Lithium brine production, reserves, resources and exploration in Chile: An updated review. Ore Geology Reviews *128*, 103883. https://doi.org/10.1016/j.oregeorev.2020.103883.

S41. Water, A., and Worley (2022). Definitive feasibility study update, Mineral Salar Blance - Lithium Project Stage one III region, Chile. https://lithiumpowerinternational.com/wp-content/uploads/2022/01/NI43-101_DFS2022_LR.pdf.

S42. Zijin Mining (n.d.). Lakkor Tso Lithium project. https://www.zjky.cn/global/program-detail-71783.htm.

S43. SRK (2023). SEC Technical Report Summary Pre-Feasibility Study Silver Peak Lithium Operation Nevada, USA. https://www.sec.gov/Archives/edgar/data/915913/000091591323000037/exhibit9631231202210-k.htm#i2ab4d569b3ee4e029d9d2ae1c46da4f8_7.

S44. Tawana (2017). Bald Hill Lithium - Tantalum Project to Deliver Outstanding Cash Flows and Returns. https://announcements.asx.com.au/asxpdf/20170711/pdf/43kkjk14z4kr79.pdf.

S45. Liontown (2021). Mineral Resurces, Reserves and CP statements. https://www.ltresources.com.au/wp-content/uploads/2023/05/624aa87d3dd52088027b8837_Resources-Reserves-and-CP-Statements.pdf.

S46. IGO (2023). Greenbushes CY23 Resources and Reserves. https://www.igo.com.au/site/pdf/483721a4-afe7-4723-a907-6efce0c66748/Greenbushes-CY23-Resources-and-Reserves.pdf.

S47. Liontown (2021). Kathleen Valley DFS confirms Tier-1 global lithium project with outstanding economics and sector-leading sustainability credentials. https://wcsecure.weblink.com.au/pdf/LTR/02450567.pdf.

S48. Allkem (2023). SEC Technical Report Summary Mt Cattlin Lithium Project. https://s203.q4cdn.com/709125885/files/doc_downloads/TechnicalRep/New/Mt-Cattlin-Lith

ium-Project-Australia.pdf.
S49. SQM (2022). Technical Report Summary Mt. Holland Lithium project. https://www.sec.gov/Archives/edgar/data/909037/000110465922049694/tm2210998d1_ex96-2.htm.
S50. Pilbara Minerals (2023). Substantial 109Mt Mineral Resource increase to 414Mt - further extends Pilgangoora's position as a world class lithium project. https://minedocs.com/24/Pilgangoora-MR-08072023.pdf.
S51. Mineral Resources (2022). Lithium Mineral Resources and Reserve Update. https://minedocs.com/23/Mineral_Resources_MR_10072022.pdf.
S52. European Lithium (2023). Wolfsberg Lithium Project Definitive Feasibility Study Results. https://minedocs.com/23/Wolfsberg-FS-03082023.pdf.
S53. Lithium Ionic (2023). Lithium Ionic Announces PEA and Expanded Mineral Resource Estimate for Bandeira; Post-tax NPV8% US$1.6 Billion & IRR of 121%. https://www.lithiumionic.com/_resources/news/231019-2.pdf.
S54. Sigma (2022). Grota Do Cirilo Lithium Project. https://minedocs.com/19/Grota-do-Cirilo-Xuxa-FS-Barreiro-PFS-5302022.pdf?utm_source=chatgpt.com.
S55. AMG (2017). AMG Lithium & Tantalum. https://amg-nv.com/wp-content/uploads/AMG-Lithium-Tantalum.pdf.
S56. Sayona (2019). Authier Lithium Project Update Definitive Feasibility Study https://www.ree.environnement.gouv.qc.ca/dossiers/3211-16-020/3211-16-020-16.pdf.
S57. RockTech (2022). Rock Tech Lithium completes Pre-Feasibility Study for its Georgia Lake Project https://res.cloudinary.com/rocktech/image/upload/v1668567754/M_Info_PFS_Summary_Release_ENG_FINAL_1ad1f00e98.pdf.
S58. SLR, WAVE, and WSP (2023). SEC Technical Report Summary Allkem Limited James Bay Lithium Project. https://s203.q4cdn.com/709125885/files/doc_downloads/TechnicalRep/New/James-Bay-Lithium-Project-Canada.pdf.
S59. Sayona (2024). Moblan Lithium Project Definitive Feasibility Study: Positive Results Deliver C$2.2B NPV. https://clients3.weblink.com.au/pdf/SYA/02774427.pdf.
S60. Sayona (2023). Definitive Feasibility Study Confirms NAL Value with A$2.2B NPV. https://wcsecure.weblink.com.au/pdf/SYA/02654503.pdf.
S61. CriticalElements (2023). Critical Elements Lithium Announces New Positive Feasibility Study for the Rose Lithium Project Generating an After-Tax NPV8% of US$2.2B and an After-Tax IRR of 65.7%. https://www.cecorp.ca/wp-content/uploads/2022-08-29-news-release-CRE.pdf.
S62. SnowLake (2024). Building a Clean Energy Company. https://snowlakeenergy.com/wp-content/uploads/2024/03/Snow-Lake-Corporate-Deck-March-2024.v1.pdf.
S63. BBA, DRA, SGS, and WSP (2023). SEC Technical Report Summary Pre-Feasibility Study on the Whabouchi mine Nemaska, Quebec. https://s203.q4cdn.com/709125885/files/doc_downloads/TechnicalRep/Nemaska-Whabouchi-Mine-Canada.pdf.

S64. Sen, N. (2024). 四川探明近百万吨锂资源量，盛新锂能参股，探转采工作正推进 (in Chinese). https://www.thepaper.cn/newsDetail_forward_26057778.

S65. Xu, S., and Wen, D. (2024). 直击股东大会丨李家沟项目采矿系统试生产 川能动力董事长：有充分信心为股东带来持续回报 (in Chinese). https://news.qq.com/rain/a/20240518A04ZK000.

S66. Zijin Mining (n.d.). Xiangyuan Hard-Rock Lithium-containing Polymetallic Mine. https://www.zijinmining.com/global/program-detail-71786.htm.

S67. Chengxin Lithium Energy (2023). 盛新锂能集团股份有限公司 2022 年年度报告 (in Chinese). https://file.finance.sina.com.cn/211.154.219.97:9494/MRGG/CNSESZ_STOCK/2023/2023-3/2023-03-07/8868659.PDF.

S68. Yichun Gov (2021). 宜春市矿产资源总体规划（2021-2025 年）（in Chinese）. https://www.doc88.com/p-74487196273821.html.

S69. SKR (2023). Keliber Lithium Project, Finland Technical Report Summary https://minedocs.com/26/Keliber_Lithium_Project_TRS_12312022.pdf.

S70. Atlantic (2023). Definitive Feasibility Study. https://minedocs.com/25/Ewoyaa_FS_06292023.pdf.

S71. Firefinch (2021). Goulamina Lithium Project Update to DFS Delivers NPV of A$4.1 Billion and 83% IRR. https://minedocs.com/22/Goulamina-FS-12062021.pdf.

S72. Lepidico (2019). Alvarrões assays indicate larger lithium Resource https://cdn.lepidico.com/production/LPD_190308_Alvarroes_assays_indicate_larger_lithium_Resource_be5f31182d.pdf.

S73. Savannah (2025). The Barroso Lithium Project: Developing Europe’s largest spodumene project. https://www.savannahresources.com/media/4ljdtggi/savannah-presentation.pdf.

S74. Piedmont (2021). Piedmont Completes Bankable Feasibility Study of the Carolina Lithium Project With Positive Results. https://www.listcorp.com/asx/pll/piedmont-lithium-limited/news/piedmont-completes-bfs-of-the-carolina-lithium-project-2647099.html.

S75. Prospect Resources (2021). Lycopodium completes Direct OFS for Arcadia Project https://prospectresources.com.au/wp-content/uploads/2022/07/61068491.pdf.

S76. ChengXin Lithium (n.d.). Mine in Africa (Sabi Star Mine). https://en.cxlithium.com/product/45.html.

S77. E3 Lithium (2024). Clearwater Project NI 43-101 Technical Report on Pre-Feasibility Study https://minedocs.com/27/Clearwater-PFS-06202024.pdf.

S78. European Metals (2022). PFS Update Delivers Outstanding Results 75% Increase in Cinovec NPV to US$1.94B 16% Increase in Production to 29,386 TPA. https://api.investi.com.au/api/announcements/emh/7381a65f-d0b.pdf.

S79. Snowden Optiro (2025). Report for Zinnwald Lithium plc & Zinnwald Lithium GmbH https://zinnwaldlithium.com/wp-content/uploads/2025/03/167-RPT-GEN-019_Rev0.pdf.

S80. Infinity (2021). San Jose Lithium Project. https://wcsecure.weblink.com.au/pdf/INF/02435564.pdf.

S81. Ausenco (2018). Technical Report on the Feasibility Study for the Sonora Lithium Project, Mexico. https://bacanoralithium.com/_userfiles/pages/files/documents/bacanorafstechnicalreport25

012018_compressed.pdf.
S82. Pacific, D. (2024). Falchani Lithium Project NI 43-101 Technical Report Preliminart Economic Assessment - Update. https://americanlithiumcorp.com/wp-content/uploads/2024/02/GPERPPR7027-Falchani-Update-of-PEA-Final-Report-22022024.pdf.
S83. Ergo (2023). An economic impact assessment of the Jadar lithium-borates project. https://ergostrategygroup.com/wp-content/uploads/2023/08/Ergo-Strategy-Group-Jadar-Economic-Impact-Assessment-Sep23-EN-spread.pdf.
S84. Fayram, T.S., Lane, T.A., and Kalmbach, D.W. (2020). NI 43-101 Technical Report Prefeasibility Study Clayton Valley Lithium Project. https://minedocs.com/20/Clayton_Valley_(Cypress)_PFS_05192020.pdf.
S85. Noram Lithium (2021). Updated Lithium Mineral Resource Estimate Zeus Project. https://noramlithiumcorp.com/site/assets/files/3886/2021-09-21-ni-43-101-report.pdf.
S86. AmericanPacific (2018). Updated Lithium Mineral Resource Estimate Zeus Project, Clayton Valley Esmeralda County, Nevada, USA. https://announcements.asx.com.au/asxpdf/20181217/pdf/441926y31gw1v9.pdf.
S87. LithiumAmericas (2022). Feasibility Study National Instrument 43-101 Technical Report for the Thacker Pass Project Humboldt County, Nevada, USA. https://minedocs.com/19/Thacker-Pass-FS-11022022.pdf.
S88. Anson Resources (2022). Paradox Lithium Project, USA Definitive Feasibility Study Presentation. https://company-announcements.afr.com/asx/asn/8b826134-2fe3-11ed-918b-c2e20985e019.pdf.
S89. Fluor (2022). IONEER USA Corp. Rhyolite Ridge Lithium-Boron Project Definitive Feasibility Study (DFS) Report https://www.ioneer.com/wp-content/uploads/2022/07/300420-dfs-executive-summary-metric_final.pdf.
S90. Standard Lithium (2023). Standard Lithium Announces Positive Preliminary Feasibility Study Results for Its South West Arkansas Project. https://minedocs.com/25/Standard-Lithium-South-West-Arkansas-PFS-PR-08082023.pdf.
S91. CAT (2022). Kamativi Lithium Project Update. https://webfiles.thecse.com/2022.10.20_NR_-_CAT_Update_on_Kamativi_Project.pdf?hF5rpznVts74PwRVnCECB11FSizA82vI.
S92. S&P. (n.d.). Mine Economics
S93. Milei, J.G., Caputo, L.A., Lucero, L.E., Thiem, M.R., González, J.M., and Hereñú, C. (2025). Portfolio of Lithium. Secretaría de Minería. https://www.argentina.gob.ar/sites/default/files/portfolio_lithium_2025.pptx.pdf.
S94. Vulcan Energy (2023). Vulcan Zero Carbon Lithium™ Project Phase One DFS results and Resources-Reserves update. https://announcements.asx.com.au/asxpdf/20230213/pdf/45ljfy3ycb8t4t.pdf.
S95. Johansen, I. (2018). Scenario modelling with morphological analysis. Technological Forecasting and Social Change *126*, 116-125.
S96. Kosow, H., and Gaßner, R. (2008). Methods of future and scenario analysis: overview, assessment, and selection criteria (DEU).

S97. Watson, J., Ketsopoulou, I., Dodds, P., Chaudry, M., Tindemans, S., Woolf, M., and Strbac, G. (2018). The security of UK energy futures.

S98. Miatto, A., Wolfram, P., Reck, B.K., and Graedel, T.E. (2021). Uncertain future of American lithium: a perspective until 2050. Environmental Science & Technology *55*, 16184-16194.

S99. Sun, X., Hao, H., Geng, Y., Liu, Z., and Zhao, F. (2024). Exploring the potential for improving material utilization efficiency to secure lithium supply for China's battery supply chain. Fundamental Research *4*, 167-177. https://doi.org/10.1016/j.fmre.2022.12.008.

S100. Martin, G., Rentsch, L., Höck, M., and Bertau, M. (2017). Lithium market research–global supply, future demand and price development. Energy Storage Materials *6*, 171-179.

S101. Speirs, J., Contestabile, M., Houari, Y., and Gross, R. (2014). The future of lithium availability for electric vehicle batteries. Renewable and Sustainable Energy Reviews *35*, 183-193.

S102. Calderon, J., Smith, N., Bazilian, M., and Holley, E. (2024). Critical mineral demand estimates for low-carbon technologies: What do they tell us and how can they evolve? Renewable and Sustainable Energy Reviews *189*, 113938.

S103. Vega-Muratalla, V.O., Ramírez-Márquez, C., Lira-Barragán, L.F., and Ponce-Ortega, J.M. (2024). Review of lithium as a strategic resource for electric vehicle battery production: Availability, extraction, and future prospects. Resources *13*, 148.

S104. Busch, P., Chen, Y., Ogbonna, P., and Kendall, A. (2025). Effects of demand and recycling on the when and where of lithium extraction. Nature Sustainability, 1-11.

S105. Jones Jr, E.C. (2024). Lithium supply chain optimization: A global analysis of critical minerals for batteries. Energies *17*, 2685.

S106. Bajolle, H., Lagadic, M., and Louvet, N. (2022). The future of lithium-ion batteries: Exploring expert conceptions, market trends, and price scenarios. Energy Research & Social Science *93*, 102850.

S107. Davis, S.J., Lewis, N.S., Shaner, M., Aggarwal, S., Arent, D., Azevedo, I.L., Benson, S.M., Bradley, T., Brouwer, J., Chiang, Y.-M., et al. (2018). Net-zero emissions energy systems. Science *360*, eaas9793. doi:10.1126/science.aas9793.

S108. Gong, H., and Andersen, A.D. (2024). The role of material resources for rapid technology diffusion in net-zero transitions: Insights from EV lithium-ion battery Technological Innovation System in China. Technological Forecasting and Social Change *200*, 123141. https://doi.org/10.1016/j.techfore.2023.123141.

S109. Knehr, K.W., Kubal, J.J., Nelson, P.A., and Ahmed, S. (2022). Battery Performance and Cost Modeling for Electric-Drive Vehicles (A Manual for BatPaC v5.0). Argonne National Laboratory (ANL), Argonne, IL (United States). https://www.osti.gov/biblio/1877590
